# Generation of Single-Cell Transcript Variability by Repression

**DOI:** 10.1016/j.cub.2017.05.028

**Published:** 2017-06-19

**Authors:** Vlatka Antolović, Agnes Miermont, Adam M. Corrigan, Jonathan R. Chubb

**Affiliations:** 1 Laboratory for Molecular Cell Biology and Division of Cell and Developmental Biology, University College London, Gower Street, London WC1E 6BT, UK

**Keywords:** stochastic gene expression, transcription, RNA stability, single-cell transcriptomics, *Dictyostelium*, heterogeneity, stochastic differentiation, self-organization, repression

## Abstract

Gene expression levels vary greatly within similar cells, even within clonal cell populations [1]. These spontaneous expression differences underlie cell fate diversity in both differentiation and disease [2]. The mechanisms responsible for generating expression variability are poorly understood. Using single-cell transcriptomics, we show that transcript variability emerging during *Dictyostelium* differentiation is driven predominantly by repression rather than activation. The increased variability of repressed genes was observed over a broad range of expression levels, indicating that variability is actively imposed and not a passive statistical effect of the reduced numbers of molecules accompanying repression. These findings can be explained by a simple model of transcript production, with expression controlled by the frequency, rather than the magnitude, of transcriptional firing events. Our study reveals that the generation of differences between cells can be a direct consequence of the basic mechanisms of transcriptional regulation.

## Highlights

• Transcript variability during development is generated predominantly by repression
• Repression-derived variability can arise from frequency modulation of transcription
• Sub-populations of undifferentiated cells are primed to express developmental genes

## Teaser

The differences between cell types that arise during development are usually assumed to result from the genes that are activated in specific lineages. In this study, Antolović et al. show, using single-cell transcriptomics, that differences between cells are generated predominantly by genes that are repressed, not those that are activated.

## Results and Discussion

**Figure 1:**
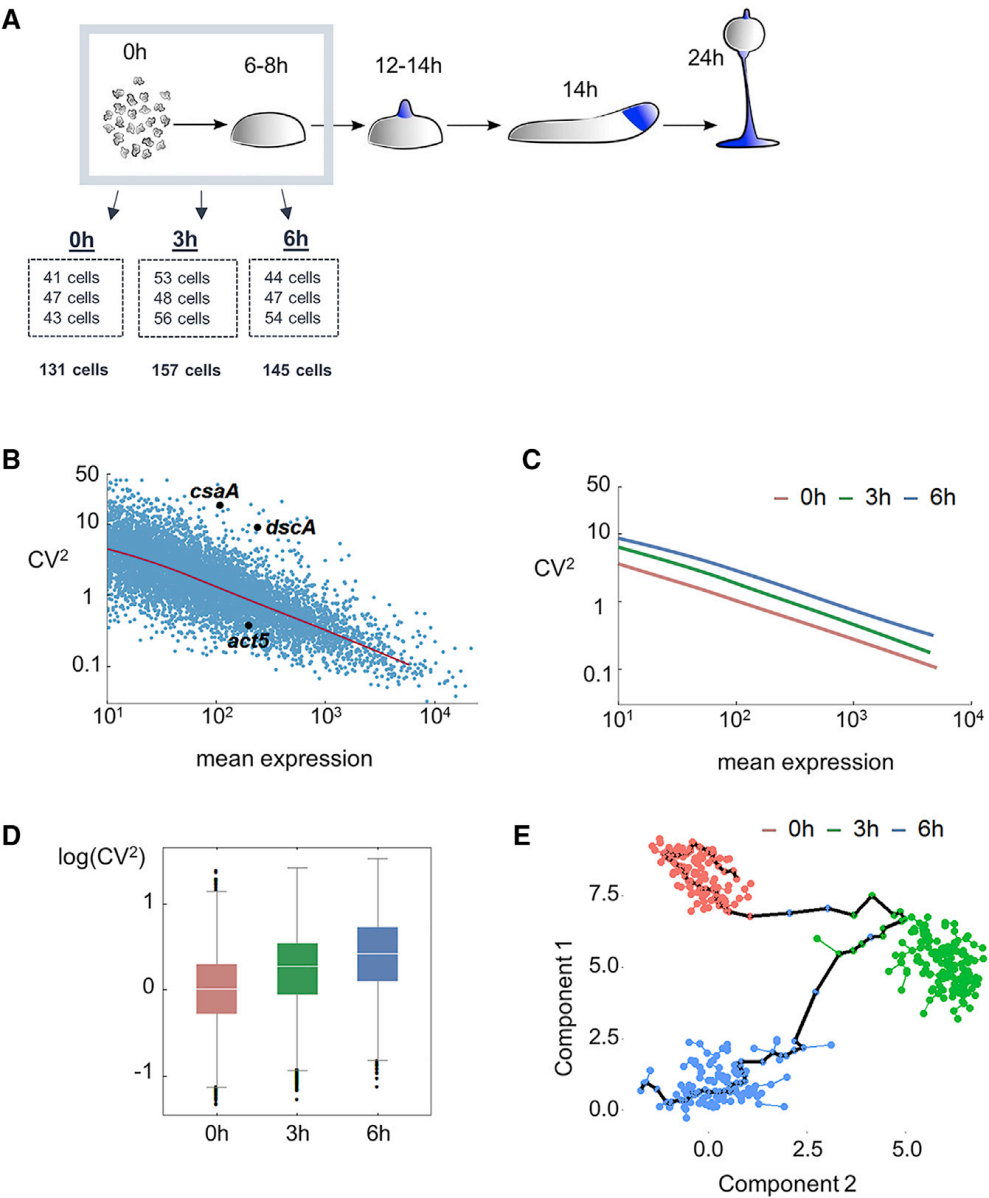
Dynamics of Gene Expression Heterogeneity during Early *Dictyostelium* Differentiation (A) Single-cell RNA-seq was carried out on 0-, 3-, and 6-hr differentiated cells. Three replicates were carried out at each stage. (B) The relationship between variance (CV^2^) and mean (read counts) of transcript levels in single 0-hr cells. The 7,670 genes (dots) with more than ten mean counts per cell are shown, with a running median in red. (C) Global noise increases during development. Data show the running medians from the three time points, averaged over all replicates. (D) The CV^2^ distribution for each time point is shown as box-and-whiskers plots, with the white line denoting the median. (E) No branches in developmental trajectories were detected by Monocle. Cells, colored by time point, are shown in the first two components’ space attained by independent component analysis. The black line shows the longest identified path through the minimal spanning tree. See also Figure S1 and Data S1.

To determine the regulatory processes underlying the generation of transcript variability, we quantified single-cell transcriptomes at multiple stages during the early differentiation of *Dictyostelium.* We sequenced the transcriptomes of 433 cells over three time points: 0 (undifferentiated cells), 3, and 6 hr (at the onset of multi-cellularity) (Figure 1A) in triplicate. Our data reproduce the expression profiles of well-studied differentiation genes in *Dictyostelium* [3] (Figure S1A).

To visualize transcript variability, the transcript variance (CV^2^, the squared coefficient of variation) was plotted against the mean expression (Figure 1B). Each gene was represented by a dot, giving a characteristic cloud showing the mean and variance of each transcript. Genes above the median line (red) were more variable than average, whereas genes below the line were less variable. The *dscA* and *csaA* transcripts showed high variability. Both genes have been shown to have highly variable protein expression [4, 5]. In contrast, actin (*act5*) showed low variability. Previous *act5* data indicate little variability, with most cells transcribing the gene at high frequency [6, 7].

Global transcript variability increases during differentiation; the whole gene cloud displayed in Figure 1B shifted vertically (Figures 1C and 1D). This increased variability occurred before branching of cells into different developmental lineages. To test for branching, we used pseudotime approaches developed for detecting bifurcations in developmental trajectories. The first method, Monocle [8], detected no branching of the developmental trajectory (Figure 1E), despite reliably ordering cells by well-known developmental markers (Figure S1B). Alternative pseudotime methods, SCUBA [9] and Wishbone [10], also did not consistently identify branching (Figures S1C and S1D). In addition, no clear segregation of cells into the primary lineages (prestalk and prespore) could be detected in correlation heatmaps of lineage markers (Figure S1E). Increased transcript variability before lineage branching has recently been observed in culture models of vertebrate hematopoiesis [11, 12] and in the early human embryo [13]. The similar behavior we have observed in the evolutionarily distinct *Dictyostelium* model suggests that this is a conserved feature of cell decision-making.

**Figure 2:**
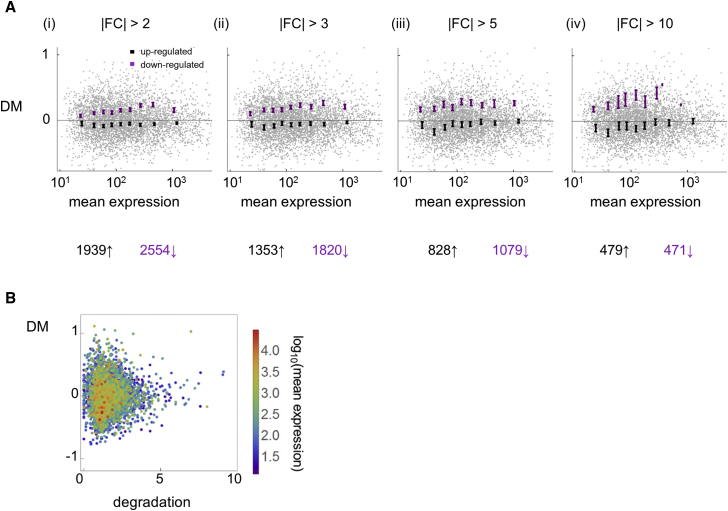
Downregulated Genes Show Greater Transcript Variability than Upregulated Genes Variability is described by DM, the deviation from the expected noise value for a given expression level [14]. See also Figures S2 and S3 and Tables S1 and S2. (A) Downregulated genes are more variable than upregulated genes. Plots show DM versus expression for up- and downregulated genes (black and purple, respectively) at 6 hr development. Data are shown for different thresholds of fold change (|FC|) in expression level of each gene between 0 and 6 hr, averaged over three replicates. Bin borders are every 500 genes within the entire dataset, starting from a mean of ten counts. Mean and SEM within each bin are shown. Numbers of up- and downregulated genes for each threshold are shown below. (B) No correlation between RNA stability and gene expression variance. Expression variability in 6-hr cells is plotted against RNA turnover (Pearson r = −0.009). Each dot represents a gene colored by its mean expression level. Degradation units are the ratio of expression before to the expression after 1-hr actinomycin D treatment [7].

During differentiation, fewer genes were upregulated than downregulated (Figure S2A), indicating the transcriptome became progressively less complex, with a greater proportion of the transcripts arising from fewer, strongly induced genes. How do up- and downregulation contribute to overall transcript diversity? To address this, we compared the transcript variability (DM, the distance to median variance [14]) of genes that were up- or downregulated at least 2-fold between 0 and 6 hr (Figure 2A). Across all levels of expression, in 6-hr cells, downregulated genes (purple) showed a greater variance (higher DM) than upregulated genes (black). Repeating the analysis, with higher fold-change thresholds in expression, showed an increasing separation between up- and downregulated genes, with the downregulated genes consistently more variable. This effect was not dependent on bin size (Figure S2B) and was also clearly apparent in the unprocessed CV^2^ values (Figure S2C). A non-parametric test revealed that the difference was highly significant (Mann-Whitney, p = 9.2 × 10^−42^).

However, a more detailed analysis was required, because downregulated genes tended to have lower read counts than upregulated genes, so that, based on the mean-variance trend in the gene cloud (Figure 1B), a lower expressed gene would likely be more variable by default. We therefore bootstrapped the data within bins of the same expression range, to determine the probability that picking randomly selected genes would generate the observed differences between up- and downregulated genes by chance: the null hypothesis that the up- and downregulated genes showed the same variability was rejected at all expression levels (p < 0.01 in each bin). The higher variability of downregulated genes was, therefore, independent of expression level. This indicates that the passive stochastic explanation— that repressed genes are more variable simply because of a statistical effect of lower numbers of molecules expressed—is not sufficient to explain the data. The greater variance of downregulated genes therefore requires another explanation.

What regulatory features of up- and downregulated genes determine variability? The turnover of RNA might affect transcript variance: less stable transcripts could reveal the presence of transcriptional noise, whereas more stable transcripts could temporally average out fluctuations. Both up- and downregulated genes showed a higher turnover than the genome average (Table S2), but we observed no relationship between RNA degradation rates [7] and transcript variance (Figure 2B). This observation suggests transcriptional noise is averaged out by longer timescales of RNA turnover. Comparing transcript variance to promoter features, such as the presence of a TATA box, promoter length, and GC content, showed no strong correlations (Figure S3), although upregulated genes tended to have longer promoters and coding sequences than downregulated genes (Table S1).

**Figure 3:**
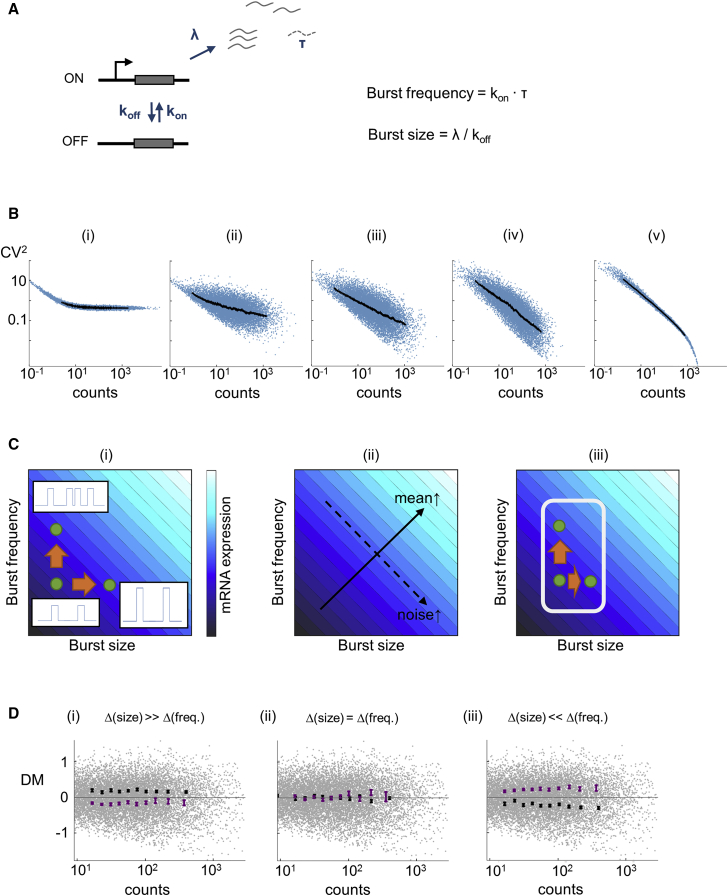
A Simple Model of Transcriptional Dynamics Explains the Global Variance Properties of Up- and Downregulated Genes (A) Two-state model of transcriptional bursting. The gene toggles between active and inactive states, with rates *k*_*on*_ and *k*_*off*_. When active, transcript production occurs at a rate λ with transcript lifetime τ. Transcript burst frequency (the frequency with which the active state occurs) is *k*_*on*_, although in most models *k*_*on*_ is scaled by τ. Burst size (the amount of RNA produced per burst) is λ/*k*_*off*_. (B) Stochastic simulation of transcription based on the model in (A) generates different simulated clouds (i–v) from different pre-set distributions of burst size and frequency (from i, where genes vary predominantly in burst size, to v, where genes vary predominantly in burst frequency, with equal contributions of size and frequency in iii). In (ii)–(iv), where both size and frequency contribute more equally, simulated data more closely resemble the experimental data. (C) Intuitive explanation of how controlling the burst parameters affects the variance of up- and downregulated genes. (i) Schematic shows mean expression is increased by increasing either burst size or frequency. (ii) Noise increases with burst size and decreases with burst frequency. (iii) Restricting the range of possible sizes and frequencies means the gene can only sample a limited range of values of mean and noise. In the example shown, the gene is mainly regulated via frequency, so an increase in expression favors a decrease in noise. (D) Matching the experimental data in Figure 2A using the two-state model. Lower expressed members of random gene pairs are more variable, if transcriptional output is determined by burst frequency rather than burst size. Shown are the simulations of randomized selections of genes constrained to have >2-fold changes in expression, allowing genes to have more variability in (i) burst size and (iii) frequency; (ii) where frequency and size vary equally. Low-expressed genes from simulated pairs (purple) and their high-expressed partners (black) are shown. Mean and SEM within each bin are shown.

Since the generation of variability by repression could not be explained in a simple manner as a passive stochastic process or by differential stability of up- and downregulated genes, this suggested a transcriptional origin for the variability. To investigate this possibility, we simulated transcript levels and variability using the two-state (or random telegraph) model of transcriptional bursting (Figure 3A) [15, 16, 17]. In this model, a gene fluctuates between an OFF state, with no transcription, and an ON state, with a certain probability of transcript production. The model allows transcriptional output to be defined in terms of transcript burst frequency (the frequency with which the ON state occurs, scaled by the RNA lifetime) and transcript burst size (the amount of RNA produced per ON phase). Both frequency and size can be modulated during normal development [7, 18].

To simulate an entire transcriptome, we specified distributions of possible values of burst frequency and size based on in vivo measurements [6, 7, 19, 20], such that the combined influence of frequency and size generated the properties of the experimental data in Figure 1B. We performed simulations with different ratios of burst size variance and burst frequency variance to the total variance in the system (Figure 3B). The distribution of simulated gene points was sensitive to the relative weights of size and frequency. The most realistic versions of the simulation occurred with strong contributions of both size and frequency to transcript level (Figure 3B, ii–iv). Extreme versions (Figure 3B, i and v), generated almost exclusively from variance in either size or frequency, did not resemble our data or data from other studies [14, 21].

To what extent can this model framework explain the expression variability of up- and downregulated genes observed in our data? Starting intuitively, transcript levels can be increased by increasing burst size, frequency, or both, so expression increases along the diagonal of a plot of frequency versus size (Figure 3C, i). Increasing burst frequency will reduce the variance in expression (due to time-averaging of noisy events), whereas increasing burst size will increase variance (due to amplification of noisy events). Therefore, the variance in expression is orthogonal to the mean in this parameter space (Figure 3C, ii). It follows that, if expression changes with a constraint on how much frequency or size can vary, this will bias the resulting variance. For example, if genes are regulated predominantly by burst frequency (box in Figure 3C, iii), then this will reduce the variance of the transcript abundance during upregulation.

To test this reasoning, we simulated the changing level of expression of a gene between two developmental time points, by randomly sampling pairs of points from the simulated cloud of genes in Figure 3B (iii). Sampling excluded pairs with less than a 2-fold difference in expression between the points, and it was weighted by the specified change in burst size and frequency. Using these pairs of points provided high and low versions of a simulated gene, giving us the opportunity to look at the overall variance characteristics of genes that have changed their expression level, based on user-defined changes in burst size, frequency, or both.

The simulations revealed differences between regulation dominated by either burst frequency or size. If regulation was equal between size and frequency, the simulation showed no difference in variance between high- and low-expressed genes of a pair (Figure 3D, ii). If regulation was dominated by burst size, the low-expressed partners showed lower variance than the high-expressed partners (Figure 3D, i). In contrast, regulation dominated by burst frequency showed that the low-expressed partners had more variability than the high-expressed partners (Figure 3D, iii).

We can interpret expression changes linking the simulated gene pairs as occurring during developmental time, with the low-expressed partners as the genes that were downregulated and the high-expressed partners as those that were upregulated. If burst size is the predominant source of regulation (Figure 3D, i), we would expect that, in differentiated cells (6 hr), the upregulated genes would be more variable. This was not observed in the experimental data (Figure 2A). If burst frequency is the dominant source of regulation (Figure 3D, iii), we would expect that downregulated genes would be more variable. This scenario matches the experimental data in Figure 2A, implying that the strong contribution of downregulation in generating expression diversity during development can be explained by the regulation of burst frequency.

Imposing down- or upregulation during development onto pairs is arbitrary; pairing only specifies a change in expression governed by a probabilistic set of rules describing the burst parameters. We could equally well interpret the simulated pairs from another point in time, such as the starting point rather than the end point. So we can use the model to predict the *initial* gene expression variance of genes that will be upregulated or downregulated by burst frequency regulation. If we consider the low-expressed partner in Figure 3D (iii) as the gene that will be upregulated by an increase in burst frequency and the high-expressed versions as the genes that will be downregulated by a decrease in burst frequency, we would expect that, in undifferentiated cells (0 hr), the genes that will later be upregulated would initially be more variable. In contrast, genes that will be later downregulated would initially be less variable. Are the experimental data consistent with these predictions?

**Figure 4:**
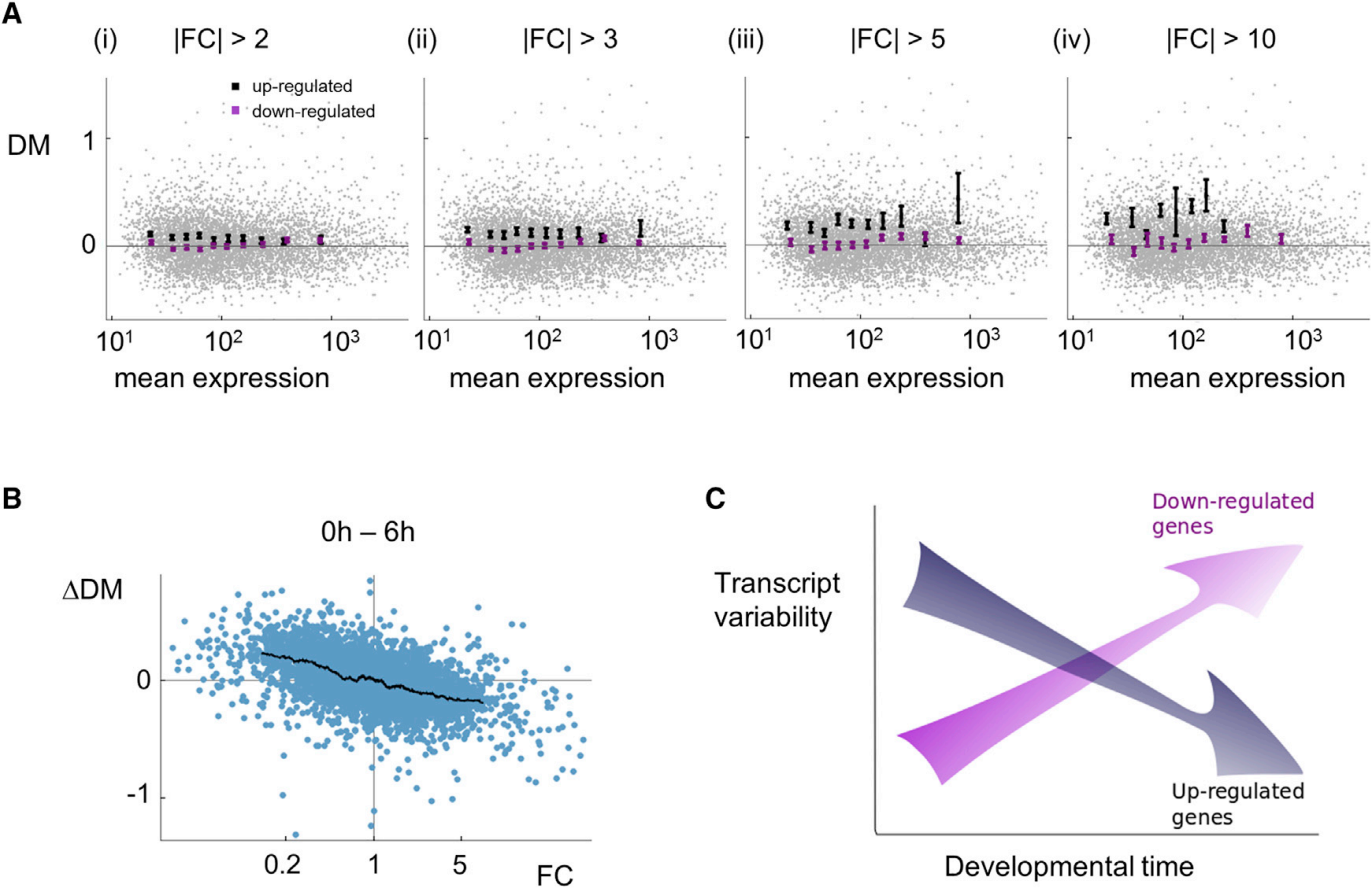
Differentiation-Induced Genes Show Elevated Transcript Variability in Undifferentiated Cells (A) Plots of variability versus expression level (read counts) for genes that will be up- and downregulated during differentiation (black and purple, respectively) before differentiation onset (0 hr). Bins are defined as in Figure 2. Mean and SEM within bins are shown for different fold-change thresholds. (B) Negative scaling of change in expression (FC) with the change in transcript variability (ΔDM) during differentiation. Variability falls in upregulated genes and increases in downregulated genes. (C) Summary. Genes induced during development are initially more variable than genes that will be repressed. Genes that are repressed become more variable than induced genes. See also Figure S4 and Data S2.

Analysis of the experimental data indicates that these predictions are valid. Figure 4A shows the plots of transcript level and variance for 0-hr cells, showing genes that will become up- or downregulated during differentiation. The genes that would be upregulated were initially more variable in their expression than those that would be downregulated. The difference is clearer for genes that undergo higher fold changes. For genes changing by 3-fold or more, for the first five bins, the difference was significant at p < 0.01 and for the next two bins at p < 0.05. This effect was apparent regardless of the bin size (Figure S4A) and was clear in the unprocessed CV^2^ values (Figure S4B). This observation might signify a knee-jerk response, of some undifferentiated cells, to the slightest hint of the differentiation trigger (starvation), and it implies that the system is geared to generate a developmentally advanced sub-population, perhaps with the potential to nucleate subsequent developmental events. In support of this idea, functional enrichment analysis indicates that starvation response genes, in addition to genes from other stress response pathways, were heterogeneously expressed in undifferentiated cells (Data S2). In contrast, the genes that were variably expressed at 6-hr development were strongly enriched for functions in several biosynthetic processes. This may relate to observations that cell fate outcome in *Dictyostelium* can be strongly influenced by the nutritional history of cells [22]. Expression distributions of selected genes following the global variance trends for up- and downregulation are displayed in Figure S4C.

Overall, our data reveal relationships between gene activation and repression and the variability in transcript levels during a developmental transition (Figures 4B and 4C). Transcript levels from genes that will be upregulated are initially more variable than those that will be downregulated. At the end of the developmental transition, transcript levels from genes that were downregulated are more variable than those from genes that were upregulated. The dynamic variability of transcripts can be explained by a simple model, in which genes are regulated by the frequency rather than the magnitude of transcriptional bursts. This view is supported by observations in multiple systems that cell signaling can regulate the frequency rather than the duration of transcriptional responses [18, 23, 24, 25]. The model cannot be expected to do justice to the full complexity of transcriptional dynamics within a burst [6], and any effects of cell-cell variation in RNA turnover will also need to be superimposed. However, the analysis represents a good first approximation, which can be compared and adapted to specific molecular effects of activation and repression during cell decision-making.

## STAR★Methods

### Key Resources Table

**Table:** 

REAGENT or RESOURCE	SOURCE	IDENTIFIER
**Critical Commercial Assays**		

Cell separation- C1 Integrated Fluidic Circuit chips	Fluidigm	100/5759, 100/5760
cDNA synthesis- Advantage 2 PCR Kit and SMARTer PCR cDNA Synthesis Kit	Clontech	200062
Library preparation- Nextera XT DNA Sample Preparation Kit	Illumina	FC-131-1096
Library preparation- Nextera Index Kit	Illumina	FC-131-1002

**Experimental Models: Organisms/Strains**		

*Dictyostelium* AX3 cells with the *rps30* gene engineered to express H2B-Cherry as a nuclear marker	[6, 25]	N/A

**Software and Algorithms**		

MATLAB R2016a	MathWorks	N/A
Mathematica 10	Wolfram	N/A
R 0.99.486	Open source	N/A
Python 3.5	Open source	N/A
Panther 11	[26]	N/A
Scuba v1.0	[9]	N/A
Wishbone	[10]	N/A
Monocle 1.4.0	[8]	N/A

### Contact for Reagent and Resource Sharing

Further information and requests for resources and reagents should be directed to and will be fulfilled by the Lead Contact, Jonathan Chubb (j.chubb@ucl.ac.uk).

### Experimental Model and Subject Details

We used *Dictyostelium* AX3 cells (mating type I). The cells had been previously engineered to express a red fluorescent nuclear marker [25]. This marker facilitated validation of single-cell capture for scRNAseq. Cells were cultured in HL5 medium attached to tissue culture dishes [6] as previously described. For development assays, cells were detached from the plastic by pipetting, washed in KK2 phosphate buffer (KPO_4_, pH 6.2) and plated on KK2/1.5% agar at a density of 3 × 10^6^ cells per 35mm dish. At the indicated times, cells were detached from the agar by gentle pipetting, and transferred in ice-cold KK2 buffer for cell capture and downstream scRNAseq processing.

### Method Details

#### Single-cell RNAseq

For single-cell RNAseq, three replicates of the developmental time series were captured. For each time point, cells were loaded onto Integrated Fluidic Circuit chips (IFC; Fluidigm). We identified capture of multiple cells and empty wells using brightfield illumination, with validation of single-cell capture initially carried out using a genetically encoded red fluorescent nuclear marker. Cell lysis, reverse transcription and cDNA pre-amplification were performed in the C1 Single-Cell Auto Prep IFC using the SMARTer PCR cDNA Synthesis Kit (Clontech) and the Advantage 2 PCR Kit, as specified by the manufacturer (protocol 100-7168 A2). ERCC RNA spike-in control mix (92 transcripts; ThermoFisher) was added to the chambers at a 1:1000 ratio. cDNA was harvested and the libraries were prepared using the Nextera XT DNA Sample Preparation Kit and the Nextera Index Kit (Illumina), according to the manufacturer’s recommendations (protocol 100-7168 A2). Libraries from one chip were pooled, and paired-end 75bp (first two replicates) or 25bp (third replicates) sequencing was performed on 4 lanes of an Illumina NextSeq500.

The quality of the reads was assessed using fastqc; a quality control tool for high throughput sequence data (www.bioinformatics.babraham.ac.uk/projects/fastqc/). Paired-end reads were mapped to the *Dictyostelium* genome (version obtained from Gareth Bloomfield, masking the duplication on chromosome 2) using Tophat version 2.0.9 (a spliced read mapper for RNA-seq; built on the mapping program Bowtie). Subsequently, we counted reads for each gene with htseq-count.

#### Stochastic simulation of transcriptional bursting

The transcription was simulated by a simple two-state model of transcriptional bursting [6], where a gene is either in the OFF state, where no transcription occurs, or in the ON state, where there is a certain probability of polymerase initiation event. The switching between the two states occurs at the rates of *k*_*on*_ and *k*_*off*_, with *k*_*off*_ being much greater than *k*_*on*_ in order for transcription to display a bursting behavior. For these simple purposes, we assume that polymerases are processive, so the rate of RNA production is equal to transcription initiation rate, λ. Once the initiation occurs, the RNA is produced with the offset of dwell time (the time needed for RNA to leave the transcription site - comprised of elongation time and termination time, and set to the physiological value of 120 s [6]). The degradation time, τ, is defined as the lifetime of RNA once it has left the transcription site.

To obtain an amount of RNAs for an individual gene in an individual cell at a certain moment in time, we performed stochastic numerical simulations, in MATLAB, of transcriptional bursting using the Gillespie algorithm [27]. The burst size, or a number of transcripts produced per burst, is defined as *λ/k*_*off*_, and the burst frequency, number of bursts per lifetime of cytoplasmic RNA, is defined as *k*_*on*_ × τ. To mimic the distributions of burst size and burst frequency, *τ and k*_*off*_ were set to constant values of 1800 *s* and 0.01 *s*^*-1*^, respectively, while λ and *k*_*on*_ were sampled from a (log_2_)-normal distribution with the mean values of 0.2 s^-1^ and 0.0013 s^-1^, respectively. Parameters set in this way result in average values of 2.34 bursts per cytoplasmic RNA lifetime and 20 RNAs synthesized during each burst. The RNA lifetime was approximated from RNA decay measurements for around 20 genes, from Northern blot data on actinomycin D treated cells [7]. The value of λ was a medium range estimate of the initiation rate from [6]. The value of *k*_*off*_ was approximated from live cell measurements of transcription for a panel of housekeeping and developmental genes, for which the majority had transcription pulses lasting less than 5 min [7]. Our estimate of *k*_*on*_ was based upon a compromise between several measurements of transcription pulse interval for different genes [7, 19, 20].

The variance of *k*_*on*_ and λ are defined through their levels of contribution to a total variance in the system, as *σ*^*2*^*(λ) = c*_*size*_ *× σ*^*2*^_*tot*_ and *σ*^*2*^*(k*_*on*_*) = c*_*freq*_ *× σ*^*2*^_*tot*_, with the total variance being *σ*^*2*^_*tot*_ *= σ*^*2*^*(λ) + σ*^*2*^*(k*_*on*_*)*. Here, *c*_*size*_ and *c*_*freq*_ are the user-specified coefficients defining the fraction of the variance contributed by burst size and frequency, respectively. To create different scenarios of how the frequency and size of transcriptional bursts vary across the genome, we set c_*size*_ and *c*_*freq*_ to the following ratios: 1:0, 0.75:0.25, 0.5: 0.5, 0.25:0.75 and 0:1. For each of these scenarios, the simulation generates 200 cells described by 12,000 randomly selected genes. For each gene, we calculated the average number of transcripts, *μ*, coefficient of variance, *CV*^*2*^, and the relative noise value, *DM*.

To simulate the process of transcriptional activation or repression, we performed a Gibbs sampling on the aforementioned simulated datasets, in order to retrieve random pairs of genes, which represent one gene either side of the developmental transition. For this purpose, we specified the probability distribution in *λ/k*_*on*_ parameter space, reflecting the user-defined probability by which the burst size and the burst frequency can change between genes in a pair. Each gene pair was selected in the following manner:

1. Randomly select the first gene *g*_*UP*_*.*
2. Repeat the following steps 1000 times:
  a. Before selecting a second gene, reject any that do not fit the two-fold expression change requirement.
  b. Assign the probability to every remaining point based on its distance from the chosen gene *g*_*UP*_ in *λ/k*_*on*_ parameter space.
  c. Randomly choose one of the points from the previous step, weighted by its probability, as a gene *g*_*DOWN*_*.*
  d. Find another *g*_*UP*_ partner by the same process described in a. to c.
3. Accept the final pair of *g*_*UP*_ and *g*_*DOWN*_*.*

Three different mechanisms of transcriptional regulation were simulated: regulation dominated by changing mostly burst frequency, regulation by changing mostly burst size or regulation by changing both properties equally. In other words, we set the values of *σ*^*2*^*(λ)* and *σ*^*2*^*(k*_*on*_*)* to satisfy one of the following conditions: *σ*^*2*^*(λ) ≪ σ*^*2*^*(k*_*on*_*)*, *σ*^*2*^*(λ) ≫ σ*^*2*^*(k*_*on*_*)* and *σ*^*2*^*(λ)* = *σ*^*2*^*(k*_*on*_*)*. A total of 2000 gene pairs were selected in this manner. Each pair contains genes with mean values at least 2 fold different. In brief, we estimated the total variance (in log_2_ space) of the experimental data as 7.3. Generating the simulated gene clouds in Figure 3B divided this variance between *σ*^*2*^*(λ) and σ*^*2*^*(k*_*on*_*)* in the aforementioned ratios- for example, for the 50:50 split, the SDs for both λ and *k*_*on*_ were 1.91, and 1.91^2^ = 3.65 = 7.3/2 (half the total variance). Using the data from the 50:50 simulation, we then set either λ or *k*_*on*_ as limiting (SD = 0.3) and the other as non-limiting (SD = 5) for setting the distributions used in the Gibbs pairing.

A key feature of the model is that genes are constrained to lie in a bounded region of burst parameter space, such that there are upper and lower limits to the burst size and frequency. This is biologically reasonable; the transcription machinery must operate within finite physical limits. This feature means that genes of the same mean expression are distributed differently in parameter space (and will have a different variance) depending on whether they are up- or downregulated.

### Quantification and Statistical Analysis

#### Analysis of read data

An average of 3 million reads were generated from each single-cell library. Raw read counts for each gene in each cell at each time point are tabulated in Data S1. We excluded cells with fewer than 500 000 and higher than 7 million reads, to exclude poor or overloaded single-cell libraries. We obtained a total of 433 cells (131 cells at 0h development, 157 cells at 3h and 145 cells at 6h). These cells also satisfied all other cell quality criteria (high number of genes detected in each cell, low percentage of mitochondrial reads and low percentage of low alignment quality reads). Reads from rRNA contaminants were excluded. Read counts of cells within each replica were normalized using the size factor from the DESeq package [28]. To minimize the impact of technical noise in our analyses, we excluded genes with a mean normalized read count < 10. For comparing the variability of up- and downregulated genes, we considered genes in bins of the same expression range, which further allows us to control for technical noise. As measures of variance we used either squared coefficient of variance $\left(CV^{2}=\sigma^{2}/\mu^{2}\right)$ or DM (distance to median CV^2^ value) [14], which accounts for the confounding effects of gene length and mean expression level on the CV^2^. Sequences and positional information of upstream intergenic regions were obtained from dictyBase [29]. TATA-containing genes were defined in [30]. RNA degradation rates were obtained from [7]. Data processing was carried out in Mathematica. For pseudotime analyses, Scuba was implemented in MATLAB, Wishbone in Python and Monocle in R. Gene Ontology enrichment analysis was performed with PANTHER Classification System version 11 [26]. GO terms enriched in both heterogeneous and homogeneous gene sets at a specific time point are excluded. Apart from the bootstrapping analysis (below), tests of significance used the non-parametric Mann-Whitney test, in Mathematica. Statistical details are provided in the relevant figure legends and manuscript text.

#### Bootstrapping

To estimate the variabilities of the calculated mean DM values of up- and downregulated genes in each bin, we performed bootstrap sampling as described [31] in Mathematica. For each bin we repeated the following procedure 10 000 times:

1. Separately resample, with replacement, upregulated and downregulated genes.
2. In each resampled set, normalize each observation’s value $\left(DM_{x}\right)$ to the same mean:

$${\tilde{DM}}_{x\in up}=DM_{x}-{\bar{DM}}_{up}+\;{\bar{DM}}_{up\cup down}$$

or

$${\tilde{DM}}_{x\in down}=DM_{x}-{\bar{DM}}_{down}+\;{\bar{DM}}_{up\cup down},$$

depending on whether the observation belongs to the up- or downregulated resampled set. In this way, we transform each sample to have a mean equal to the overall mean of the population and so generate the H_0_ hypothesis.

3. Perform the Student’s t test between the distributions of ${\tilde{DM}}_{up}$ and ${\tilde{DM}}_{down}$ to get a *t-*value, *t*_*i*_*.*

After collecting the n = 10 000 *t*_*i*_ values, we calculate the probability of *DM* values of up- and downregulated genes to be significantly different by chance as:

$$p\left(H_{0}\right)=\frac{1\;+\sum_{i=1}^{n}\left(t_{i}\;>t\right)\;}{1+\;n},$$

with *t* being the original sample *t*-value.

### Data and Software Availability

Raw read count data from the single-cell RNAseq is provided as Data S1. The MATLAB code used for the simulations is available at http://www.ucl.ac.uk/lmcb/sites/default/files/Simulation_2017.zip.

## Author Contributions

A.M. performed the experiments. V.A., A.M., and J.R.C. analyzed the data. V.A. and A.M.C. performed the modeling. All authors wrote the manuscript.

## Supplemental Information

Document S1. Figures S1–S4 and Tables S1 and S2

Data S1. Raw Read Counts

Data S2. Gene Ontology Analysis of Variable and Homogenously Expressed Genes

Document S2. Article plus Supplemental Information
