# Supplementary material for: Generation of Single-Cell Transcript Variability by Repression
Source: Curr Biol. 2017 Jun 19;27(12):1811–1817.e3. doi: 10.1016/j.cub.2017.05.028 (PMC5483230; doi:10.1016/j.cub.2017.05.028)
Supplement: Document S1. Figures S1–S4 and Tables S1 and S2 [file mmc1.pdf]

Current Biology, Volume *27*

Supplemental Information

Generation of Single-Cell Transcript Variability by Repression

Vlatka Antolović, Agnes Miermont, Adam M. Corrigan, and Jonathan R. Chubb

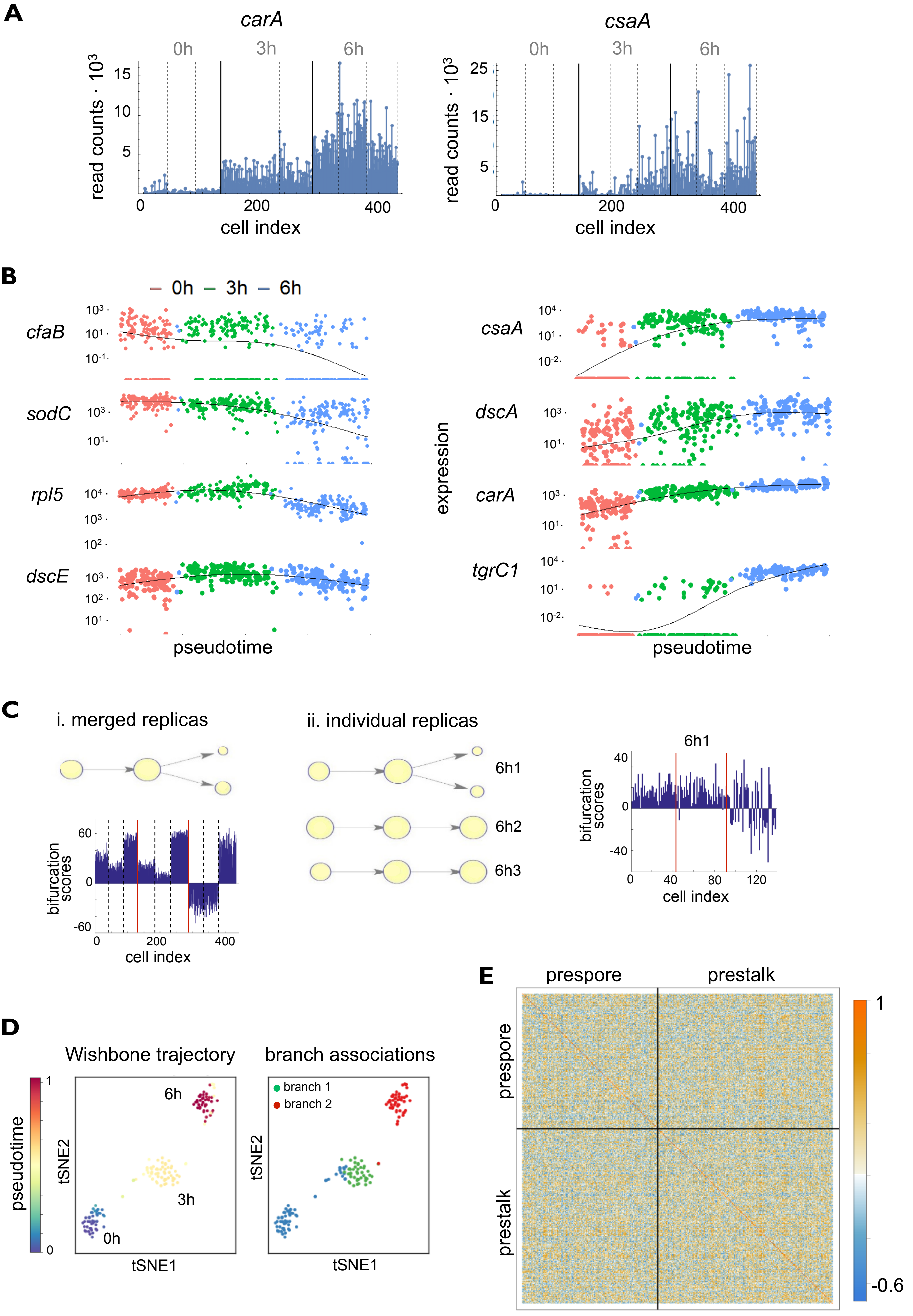

A
carA
csaA
0h
3h
6h
read counts · 10³
cell index
B
— 0h — 3h — 6h
cfaB
sodC
rpl5
dscE
csaA
dscA
carA
tgrC1
expression
pseudotime
C
i. merged replicas
ii. individual replicas
6h1
6h2
6h3
bifurcation scores
cell index
D
Wishbone trajectory
branch associations
pseudotime
branch 1
branch 2
tSNE1
tSNE2
E
prespore
prestalk

Figure S1 . Testing single cell expression analyses and cell trajectories. Related to Figure 1.

A) Expression profiles of known developmental marker genes, *carA* and *csaA* throughout development. Plots show cell read counts from each developmental time point, with the three developmental time points separated by solid vertical gridlines and each replica separated by dotted vertical gridlines. **B)** To test the validity of Monocle, we extracted the changing expression levels of well-studied developmental marker genes in pseudotime. Each dot represents a cell coloured by its developmental time point (red (0h), green (3h) and blue (6h)) and ordered along the x-axis by its assigned pseudotime, and y-axis by the expression level of the indicated gene. The black line shows a smoothed running median of gene expression levels. There is good agreement between these pseudotime-series' and published data. **C)** Bifurcation trees generated by SCUBA using either cells from all 3 replicates (i), or cells from separated replicates (ii). Bottom panel for (i) shows bar plots of the scores for each cell on the bifurcation axis (above the axis is one branch, below the axis is another). The three developmental time points are separated by vertical red lines, and replicates by vertical dotted lines. The bifurcation in i) detects the differences between replicas, not cells within a replica. Only 1/3 replicates shows a bifurcation at 6h. The bifurcation plot for (ii) is only for the replicate (6h1) showing a bifurcation. **D)** Wishbone trajectory and branch association are visualized on tSNE maps. The first two diffusion components are used, based on their highest contribution to overall population variance. When branching is imposed, the 3h and 6h time points are identified as two different developmental cell fates. The use of some higher order diffusion components can result in branching at 6h, although this result is inconsistent between replicas and may be driven mostly by the variability in the developmental progress of the early aggregative cells. **E)** No clear patterns of correlation within lineage specific genes in single cells. Correlation heatmap for the 6h timepoint (replicates pooled) split into prespore and prestalk genes. We selected cell-type specific genes from the intersection between the data sets of [S1] and DictyExpress [S2] with $|\log_2 (FC)| > 1$, $-\log_{10} (FDR) > 1$ and a maximum expression level > 100.

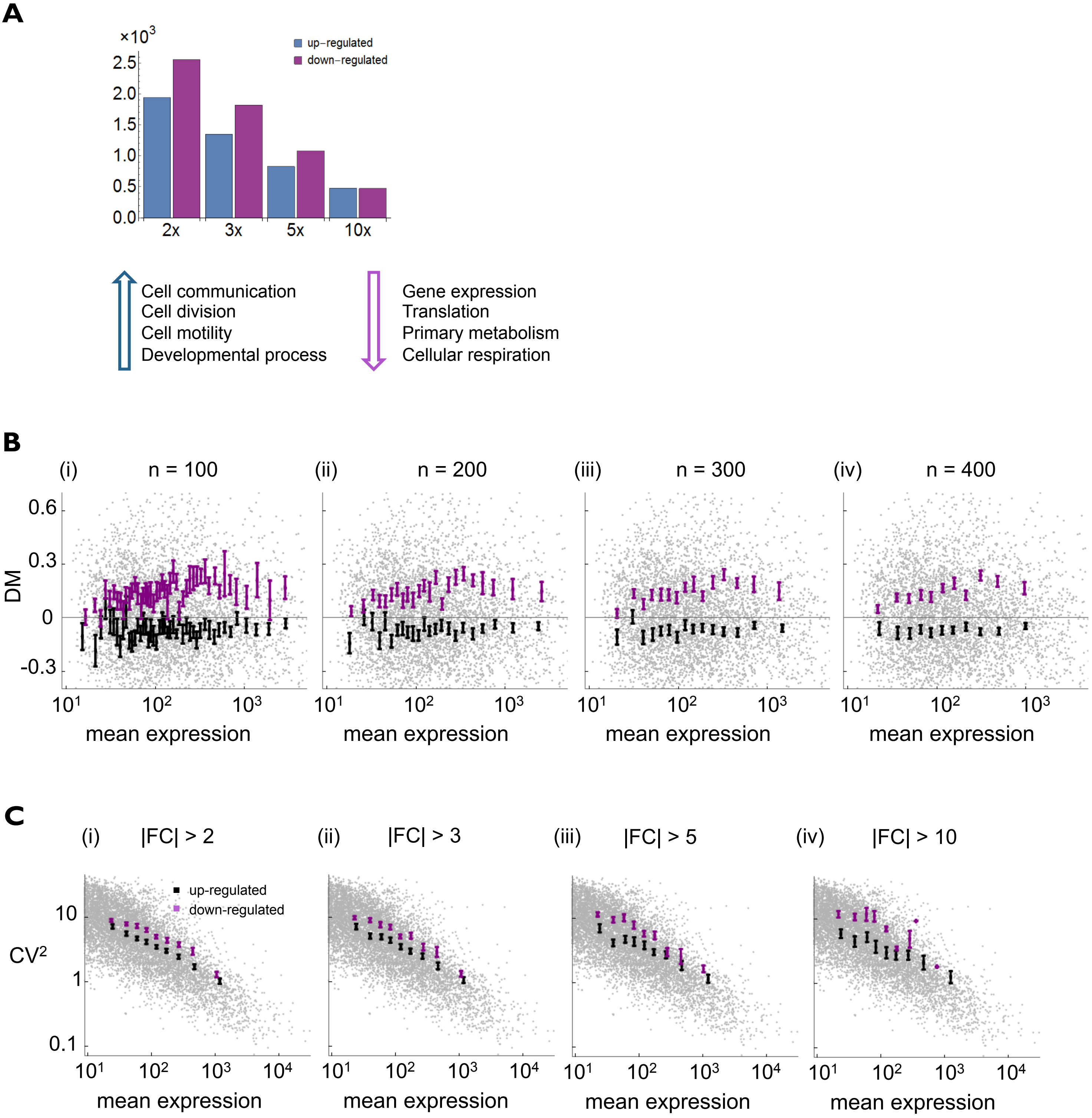
A
×10^3
2.5
2.0
1.5
1.0
0.5
0.0
2x
3x
5x
10x
up–regulated
down–regulated
Cell communication
Cell division
Cell motility
Developmental process
Gene expression
Translation
Primary metabolism
Cellular respiration
B
(i) n = 100
(ii) n = 200
(iii) n = 300
(iv) n = 400
DM
0.6
0.3
0
-0.3
10^1
10^2
10^3
mean expression
C
(i) |FC| > 2
(ii) |FC| > 3
(iii) |FC| > 5
(iv) |FC| > 10
up-regulated
down-regulated
CV^2
10
1
0.1
10^4
mean expression

Figure S2. Effects of gene down-regulation on transcript variability. Related to Figure 2.

A) More genes are repressed than activated as cells differentiate. Around 25 % more genes are down-regulated than up-regulated as cells progress from the undifferentiated state (0h) to the onset of multicellularity (6h). The numbers of up- and down-regulated genes (blue and purple, respectively) are shown for the indicated different thresholds of fold change. Data are averaged from 3 experimental replicates. **B)** Testing the independence of chosen bin size on the observation that down-regulated genes in aggregative cells (6h) are more variable than up-regulated genes. Plots of DM (variability) versus expression level (read counts) for up- and down-regulated genes (black and purple, respectively) for the threshold of fold change $|FC| > 2$. Data are shown for the bin borders defined as every 100 (i), 200 (ii), 300 (iii) or 400 (iv) genes from the whole data set, starting from mean expression value of 10. Genes that were up- and down-regulated between 0h (undifferentiated cells) and 6h development (aggregating cells) are identified, and their mean DM values and standard errors are shown within each bin at the 6h timepoint. **C)** Down-regulated genes show more transcript variability than up-regulated genes. These data are the same as in Figure 2A, using CV^2 values, rather than DM, which has been corrected for transcript size. For $|FC| > 2$, the first and last bins were significant at $p<0.05$. All other bins were significant at $p<0.001$.

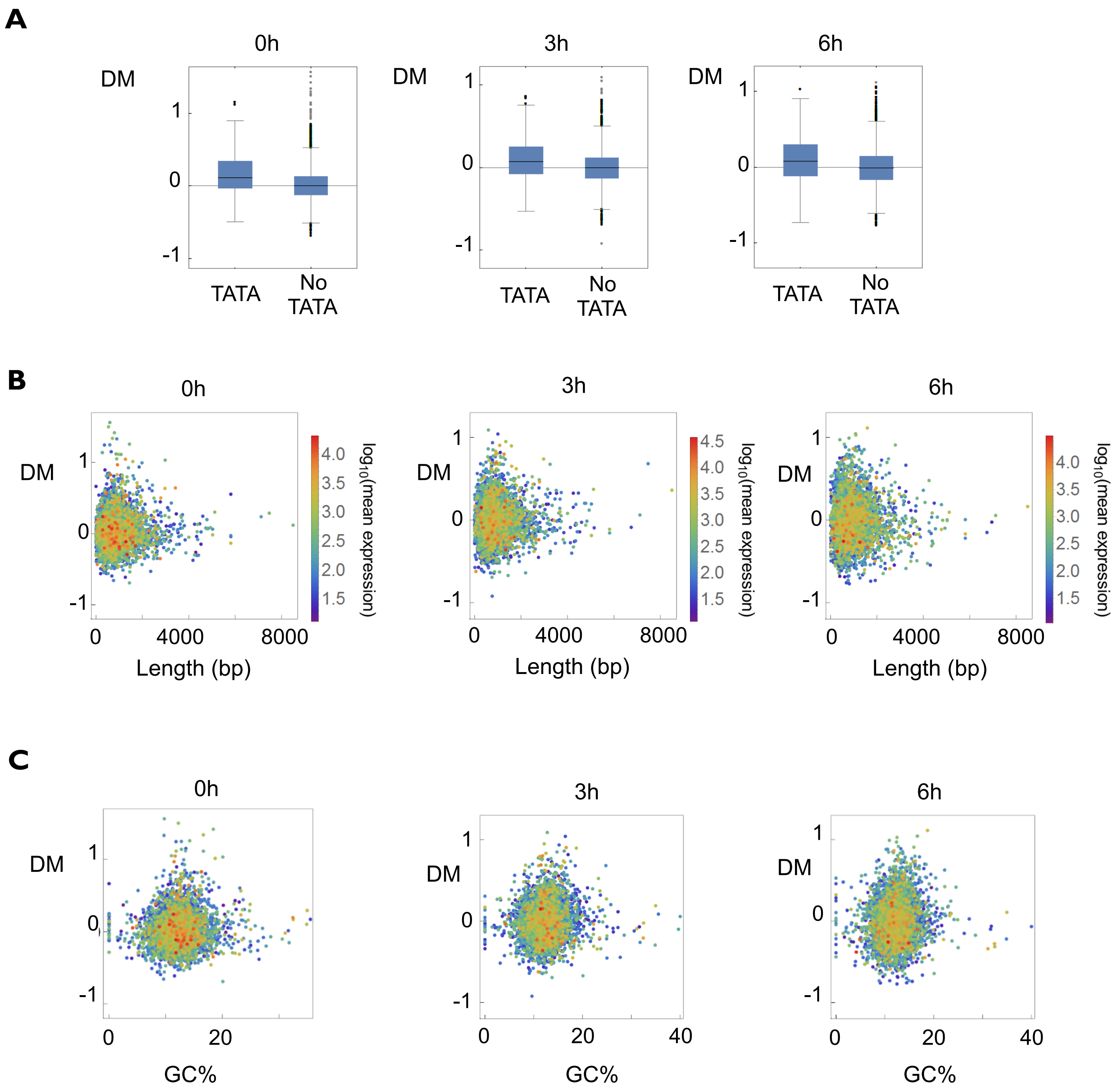
A
0h
3h
6h
DM
1
0
-1
TATA
No TATA
B
0h
3h
6h
DM
Length (bp)
0
4000
8000
log10(mean expression)
1.5
2.0
2.5
3.0
3.5
4.0
4.5
C
0h
3h
6h
DM
GC%
0
20
40

Figure S3. Promoter features and transcript diversity. Related to Figure 2.

A) Genes with an identified TATA box in the promoter region show higher expression variability. However, this effect is dependent upon correcting DM values for transcript size. TATA genes tend to be shorter than non-TATA genes in *Dictyostelium*, and in uncorrected CV^2 data, no difference is seen. Distributions of expression variability in the population of 0h cells (0h, Mann-Whitney $p= 3.9 \times 10^{-11}$), 3h differentiated cells (Mann-Whitney $p= 9.1 \times 10^{-6}$) and the aggregative cells (6h; $p= 3.4 \times 10^{-6}$) for genes with and without TATA boxes are shown. **B)** No correlation between promoter length and gene expression variability. DM plotted against length of upstream intergenic region for all three time points. Pearson r values are: 0.06 (0h), 0.03 (3h) and -0.02 (6h). Each dot represents a gene coloured by its mean expression level. **C)** No correlation between promoter GC content and gene expression variability at any time point during early development. Pearson r values: 0.08 (0h), 0.07 (3h) and 0.07 (6h). Each dot represents a gene coloured by its mean expression level.

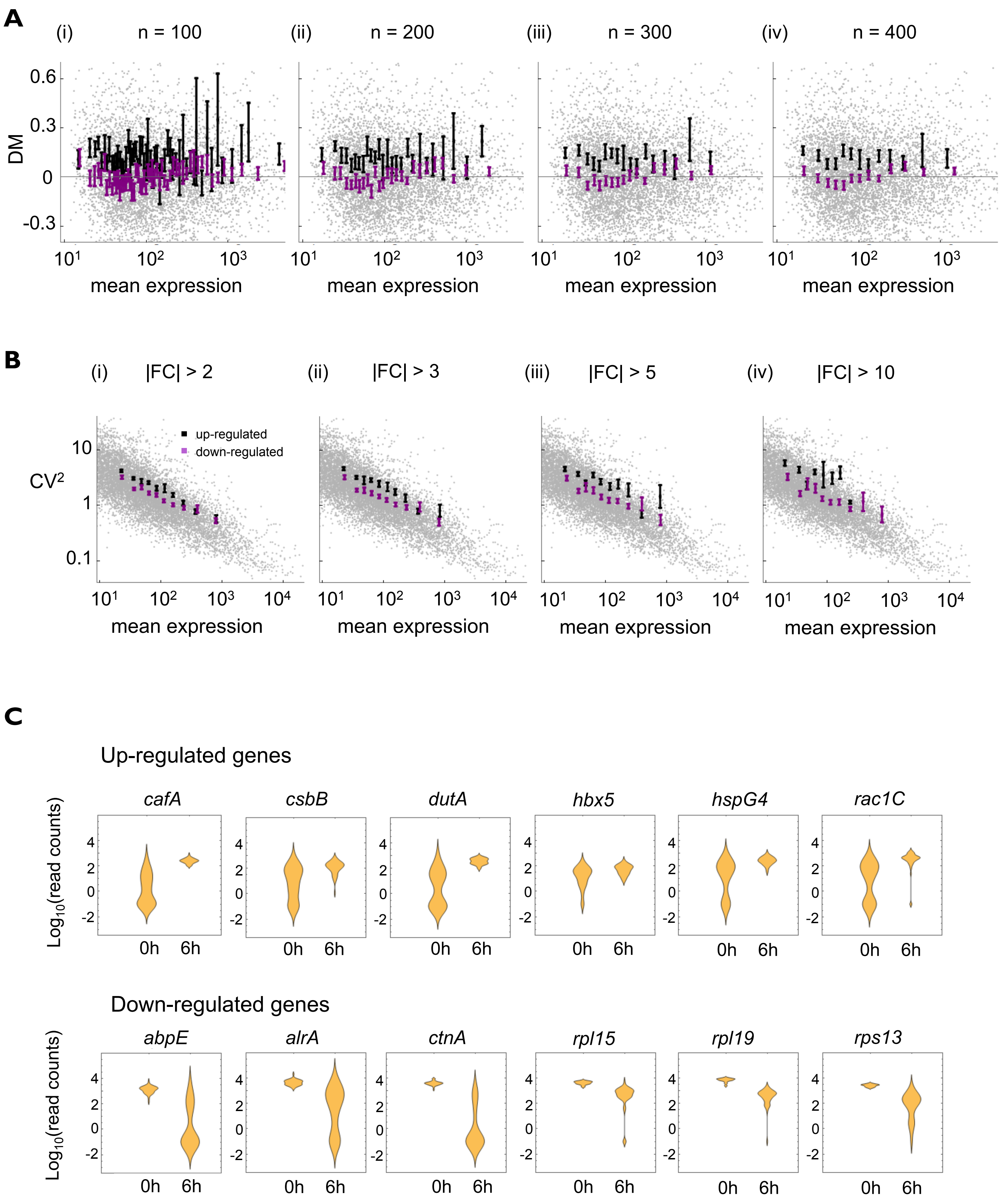
A
(i) n = 100
(ii) n = 200
(iii) n = 300
(iv) n = 400
DM
0.6
0.3
0
-0.3
mean expression
B
(i) |FC| > 2
(ii) |FC| > 3
(iii) |FC| > 5
(iv) |FC| > 10
up-regulated
down-regulated
CV2
10
1
0.1
mean expression
C
Up-regulated genes
cafA
csbB
dutA
hbx5
hspG4
rac1C
Log10(read counts)
0h
6h
Down-regulated genes
abpE
alrA
ctnA
rpl15
rpl19
rps13
Log10(read counts)
0h
6h

Figure S4. Robustness of transcript variability measurements. Related to Figure 4.

A) Testing the independence of chosen bin size on the observation that genes in undifferentiated cells (0h) that will be down-regulated are less variable than genes that will be up-regulated. Plots of DM (variability) versus expression level (read counts) for genes that will be up- and down-regulated during differentiation (black and purple, respectively) before differentiation onset (0h). Data are shown for the bin borders defined as every 100 (i), 200 (ii), 300 (iii) or 400 (iv) genes from the whole data set, starting from mean expression value of 10. **B)** Transcripts that will be up-regulated in development are initially more variable. These data are the same as in Figure 4A, using normalised CV^2 values, rather than DM, which has been corrected for transcript size. For $|FC| > 2$, the first 8 bins showed a significant difference at $p<0.001$. **C)** Transcript count distributions of selected genes representing the global transcriptome trends. Upper row- genes up-regulated during differentiation showing strongly reduced variability. Genes are *cafA* (calcium binding), *csbB* (cell adhesion), *dutA* (ncRNA), *hbx5* (homeobox transcription factor), *hspG4* (heat shock protein), *rac1C* (small GTPase). Bottom- genes down-regulated during differentiation with increased variability. Genes are *abpE* (actin binding protein), *alrA* (aldehyde reductase), *ctnA* (quorum sensing), *rpl15, rpl19 and rps13* (all ribosomal proteins).

GENE LENGTH								
Fold change threshold	All genes		Up-regulated			Down-regulated		
	**Median [IQR] (bp)**	**n**	**Median [IQR] (bp)**	**n**	**p-value**	**Median [IQR] (bp)**	**n**	**p-value**
**2**	1482 [878, 2446]	11244	1861 [1143, 3031]	1889	9.4×10^{-38}	1411 [891, 2191]	2509	9.1×10^{-3}
**3**			1839 [1106, 3012]	1315	2.6×10^{-23}	1356 [864, 2130]	1788	2.3×10^{-5}
**5**			1719 [993, 2813]	798	2.6×10^{-7}	1344 [849, 2064]	1060	3.6×10^{-5}
**10**			1689 [930, 2761]	460	2.1×10^{-3}	1365 [892, 2076]	464	1.9×10^{-2}
INTERGENIC DISTANCE								
Fold change threshold	All genes		Up-regulated			Down-regulated		
	**Median [IQR] (bp)**	**n**	**Median [IQR] (bp)**	**n**	**p-value**	**Median [IQR] (bp)**	**n**	**p-value**
**2**	678 [413, 1058]	11244	853 [517, 1325]	1889	1.7×10^{-34}	618 [387, 944]	2509	2.2×10^{-9}
**3**			871 [523, 1379]	1315	2.9×10^{-29}	620 [390, 945]	1788	7.5×10^{-7}
**5**			891 [559, 1382]	798	5.7×10^{-25}	623 [395, 981]	1060	2.0×10^{-3}
**10**			921 [585, 1423]	460	1.9×10^{-19}	640 [390, 978]	464	3.4×10^{-2}

Table S1. Comparing expression behaviour with gene length and intergenic distance. Related to Figure 2.

Distributions of gene length and intergenic distance (acquired from dictyBase) in the sets of genes detected as up- and down-regulated at different fold change thresholds are compared with the distribution of gene length and intergenic distance throughout the *Dictyostelium* genome. Mann-Whitney test *p*-values are shown. Interquartile ranges (IQR) shown in brackets.

Fold change threshold	All genes		Up-regulated			Down-regulated		
	**Median [IQR]**	**n**	**Median [IQR]**	**n**	**p-value**	**Median [IQR]**	**n**	**p-value**
**2**	1.29 [0.64, 1.96]	13210	1.48 [0.98, 2.19]	1916	1.8×10^{-28}	1.42 [1.00, 1.94]	2496	4.4×10^{-21}
**3**			1.44 [0.90, 2.20]	1335	7.5×10^{-17}	1.42 [0.99, 1.96]	1771	8.9×10^{-17}
**5**			1.35 [0.82, 2.11]	816	1.8×10^{-5}	1.43 [1.01, 2.01]	1041	3.3×10^{-14}
**10**			1.30 [0.70, 2.01]	471	0.13	1.53 [1.04, 2.12]	450	7.6×10^{-11}

Table S2. Comparing expression behaviour with RNA turnover. Related to Figure 2.

Distributions of RNA turnover for the sets of genes detected as up- and down-regulated at different fold change thresholds are compared with the distribution of RNA turnover throughout the *Dictyostelium* genome. Mann-Whitney test *p*-values are shown. Interquartile ranges shown in brackets. RNA turnover (degradation) expressed as ratio of expression before and after 1h treatment with actinomycin D.

Supplemental References

S1. Parikh, A., Miranda, E.R., Katoh-Kurasawa, M., Fuller, D., Rot, G., Zagar, L., Curk, T., Sucgang, R., Chen, R., Zupan, B., et al. (2010). Conserved developmental transcriptomes in evolutionarily divergent species. Genome biology *11*, R35.

S2. Rot, G., Parikh, A., Curk, T., Kuspa, A., Shaulsky, G., and Zupan, B. (2009). dictyExpress: a Dictyostelium discoideum gene expression database with an explorative data analysis web-based interface. BMC bioinformatics *10*, 265.
