# Supplementary material for: Generation of Single-Cell Transcript Variability by Repression
Source: Curr Biol. 2017 Jun 19;27(12):1811–1817.e3. doi: 10.1016/j.cub.2017.05.028 (PMC5483230; doi:10.1016/j.cub.2017.05.028)
Supplement: Document S2. Article plus Supplemental Information [file mmc4.pdf]

Report

Current Biology

Generation of Single-Cell Transcript Variability by Repression

Highlights

- Transcript variability during development is generated predominantly by repression
- Repression-derived variability can arise from frequency modulation of transcription
- Sub-populations of undifferentiated cells are primed to express developmental genes

Authors

Vlatka Antolović, Agnes Miermont, Adam M. Corrigan, Jonathan R. Chubb

Correspondence

j.chubb@ucl.ac.uk

In Brief

The differences between cell types that arise during development are usually assumed to result from the genes that are activated in specific lineages. In this study, Antolović et al. show, using single-cell transcriptomics, that differences between cells are generated predominantly by genes that are repressed, not those that are activated.

Antolović et al., 2017, Current Biology *27*, 1811–1817
June 19, 2017 © 2017 The Author(s). Published by Elsevier Ltd.
http://dx.doi.org/10.1016/j.cub.2017.05.028

CellPress

Generation of Single-Cell Transcript Variability by Repression

Vlatka Antolović,[1,2] Agnes Miermont,[1,2] Adam M. Corrigan,[1] and Jonathan R. Chubb[1,3,*]
[1]Laboratory for Molecular Cell Biology and Division of Cell and Developmental Biology, University College London, Gower Street, London WC1E 6BT, UK
[2]These authors contributed equally
[3]Lead Contact
*Correspondence: j.chubb@ucl.ac.uk
http://dx.doi.org/10.1016/j.cub.2017.05.028

SUMMARY

Gene expression levels vary greatly within similar cells, even within clonal cell populations [1]. These spontaneous expression differences underlie cell fate diversity in both differentiation and disease [2]. The mechanisms responsible for generating expression variability are poorly understood. Using single-cell transcriptomics, we show that transcript variability emerging during *Dictyostelium* differentiation is driven predominantly by repression rather than activation. The increased variability of repressed genes was observed over a broad range of expression levels, indicating that variability is actively imposed and not a passive statistical effect of the reduced numbers of molecules accompanying repression. These findings can be explained by a simple model of transcript production, with expression controlled by the frequency, rather than the magnitude, of transcriptional firing events. Our study reveals that the generation of differences between cells can be a direct consequence of the basic mechanisms of transcriptional regulation.

RESULTS AND DISCUSSION

To determine the regulatory processes underlying the generation of transcript variability, we quantified single-cell transcriptomes at multiple stages during the early differentiation of *Dictyostelium.* We sequenced the transcriptomes of 433 cells over three time points: 0 (undifferentiated cells), 3, and 6 hr (at the onset of multi-cellularity) (Figure 1A) in triplicate. Our data reproduce the expression profiles of well-studied differentiation genes in *Dictyostelium* [3] (Figure S1A).

To visualize transcript variability, the transcript variance (CV^2, the squared coefficient of variation) was plotted against the mean expression (Figure 1B). Each gene was represented by a dot, giving a characteristic cloud showing the mean and variance of each transcript. Genes above the median line (red) were more variable than average, whereas genes below the line were less variable. The *dscA* and *csaA* transcripts showed high variability. Both genes have been shown to have highly variable protein expression [4, 5]. In contrast, actin (*act5*) showed low variability. Previous *act5* data indicate little variability, with most cells transcribing the gene at high frequency [6, 7].

Global transcript variability increases during differentiation; the whole gene cloud displayed in Figure 1B shifted vertically (Figures 1C and 1D). This increased variability occurred before branching of cells into different developmental lineages. To test for branching, we used pseudotime approaches developed for detecting bifurcations in developmental trajectories. The first method, Monocle [8], detected no branching of the developmental trajectory (Figure 1E), despite reliably ordering cells by well-known developmental markers (Figure S1B). Alternative pseudotime methods, SCUBA [9] and Wishbone [10], also did not consistently identify branching (Figures S1C and S1D). In addition, no clear segregation of cells into the primary lineages (prestalk and prespore) could be detected in correlation heatmaps of lineage markers (Figure S1E). Increased transcript variability before lineage branching has recently been observed in culture models of vertebrate hematopoiesis [11, 12] and in the early human embryo [13]. The similar behavior we have observed in the evolutionarily distinct *Dictyostelium* model suggests that this is a conserved feature of cell decision-making.

During differentiation, fewer genes were upregulated than downregulated (Figure S2A), indicating the transcriptome became progressively less complex, with a greater proportion of the transcripts arising from fewer, strongly induced genes. How do up- and downregulation contribute to overall transcript diversity? To address this, we compared the transcript variability (DM, the distance to median variance [14]) of genes that were up- or downregulated at least 2-fold between 0 and 6 hr (Figure 2A). Across all levels of expression, in 6-hr cells, downregulated genes (purple) showed a greater variance (higher DM) than upregulated genes (black). Repeating the analysis, with higher fold-change thresholds in expression, showed an increasing separation between up- and downregulated genes, with the downregulated genes consistently more variable. This effect was not dependent on bin size (Figure S2B) and was also clearly apparent in the unprocessed CV^2 values (Figure S2C). A non-parametric test revealed that the difference was highly significant (Mann-Whitney, $p = 9.2 \times 10^{-42}$).

However, a more detailed analysis was required, because downregulated genes tended to have lower read counts than upregulated genes, so that, based on the mean-variance trend in the gene cloud (Figure 1B), a lower expressed gene would likely be more variable by default. We therefore bootstrapped the data within bins of the same expression range, to determine the

This is an open access article under the CC BY license (http://creativecommons.org/licenses/by/4.0/).

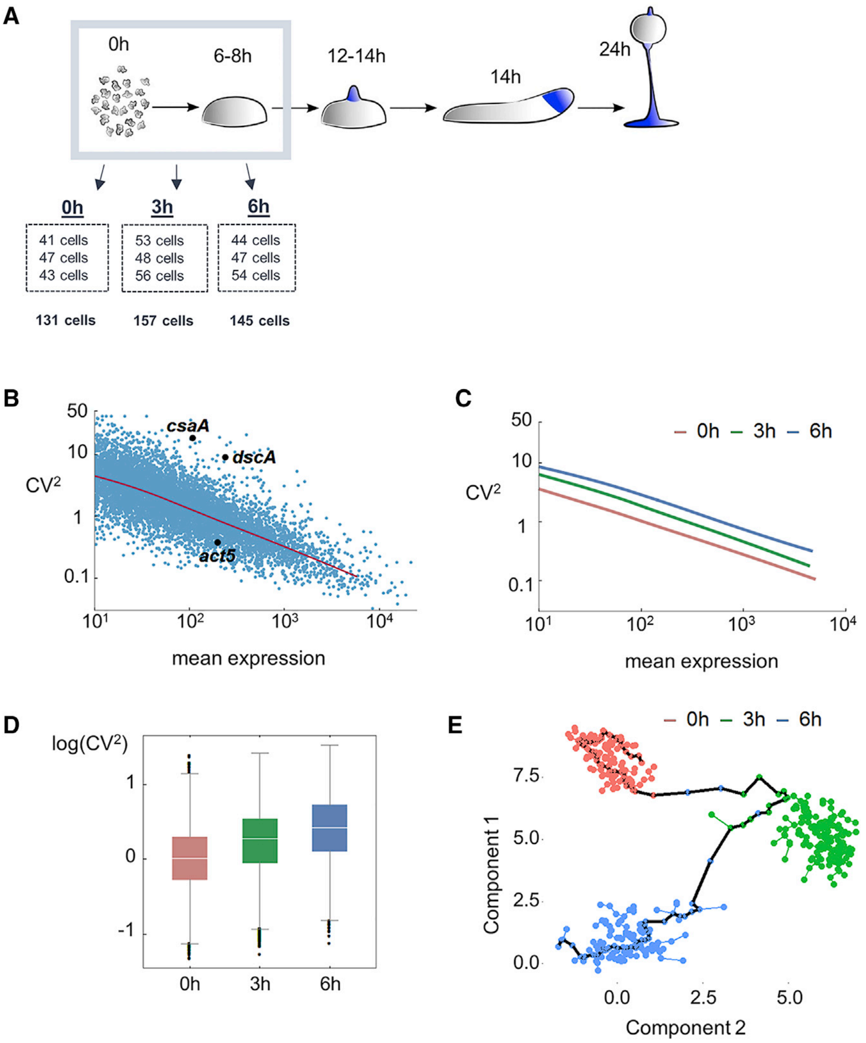

Figure 1. Dynamics of Gene Expression Heterogeneity during Early *Dictyostelium* Differentiation

(A) Single-cell RNA-seq was carried out on 0-, 3-, and 6-hr differentiated cells. Three replicates were carried out at each stage.

(B) The relationship between variance (CV^2) and mean (read counts) of transcript levels in single 0-hr cells. The 7,670 genes (dots) with more than ten mean counts per cell are shown, with a running median in red.

(C) Global noise increases during development. Data show the running medians from the three time points, averaged over all replicates.

(D) The CV^2 distribution for each time point is shown as box-and-whiskers plots, with the white line denoting the median.

(E) No branches in developmental trajectories were detected by Monocle. Cells, colored by time point, are shown in the first two components' space attained by independent component analysis. The black line shows the longest identified path through the minimal spanning tree.

See also Figure S1 and Data S1.

probability that picking randomly selected genes would generate the observed differences between up- and downregulated genes by chance: the null hypothesis that the up- and downregulated genes showed the same variability was rejected at all expression levels ($p < 0.01$ in each bin). The higher variability of downregulated genes was, therefore, independent of expression level. This indicates that the passive stochastic explanation—that repressed genes are more variable simply because of a statistical effect of lower numbers of molecules expressed—is not sufficient to explain the data. The greater variance of downregulated genes therefore requires another explanation.

What regulatory features of up- and downregulated genes determine variability? The turnover of RNA might affect transcript variance: less stable transcripts could reveal the presence of transcriptional noise, whereas more stable transcripts could temporally average out fluctuations. Both up- and downregulated genes showed a higher turnover than the genome average (Table S2), but we observed no relationship between RNA degradation rates [7] and transcript variance (Figure 2B). This observation suggests transcriptional noise is averaged out by longer timescales of RNA turnover. Comparing transcript variance to promoter features, such as the presence of a TATA box, promoter length, and GC content, showed no strong correlations (Figure S3), although upregulated genes tended to have longer promoters and coding sequences than downregulated genes (Table S1).

Since the generation of variability by repression could not be explained in a simple manner as a passive stochastic process or by differential stability of up- and downregulated genes, this suggested a transcriptional origin for the variability. To investigate this possibility, we simulated transcript levels and variability using the two-state (or random telegraph) model of transcriptional bursting (Figure 3A) [15–17]. In this model, a gene fluctuates between an OFF state, with no transcription, and an ON state, with a certain probability of transcript production. The model allows transcriptional output to be defined in terms of transcript burst frequency (the frequency with which the ON state occurs, scaled by the RNA lifetime) and transcript burst size (the amount of RNA produced per ON phase). Both frequency and size can be modulated during normal development [7, 18].

To simulate an entire transcriptome, we specified distributions of possible values of burst frequency and size based on in vivo measurements [6, 7, 19, 20], such that the combined influence of frequency and size generated the properties of the experimental data in Figure 1B. We performed simulations with different ratios of burst size variance and burst frequency variance to the total variance in the system (Figure 3B). The distribution of simulated gene points was sensitive to the relative weights of size and frequency. The most realistic versions of the simulation occurred with strong contributions of both size and frequency to transcript level (Figure 3B, ii–iv). Extreme

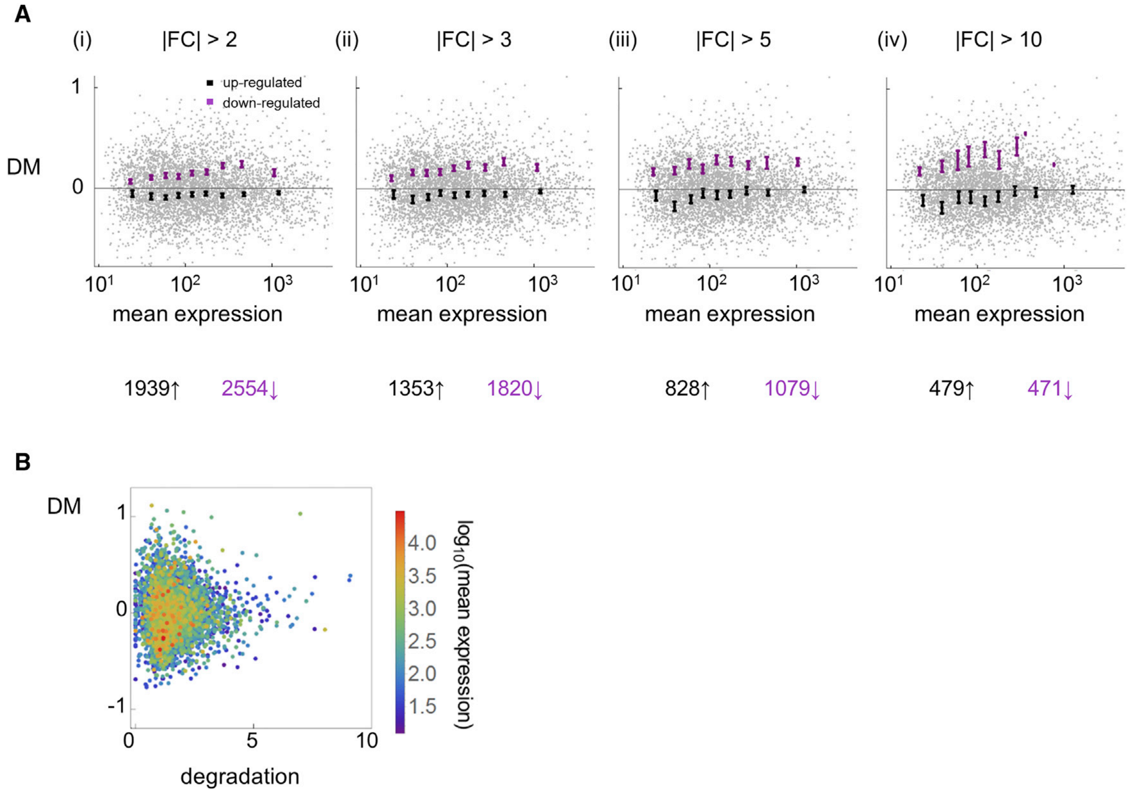

Figure 2. Downregulated Genes Show Greater Transcript Variability than Upregulated Genes

Variability is described by DM, the deviation from the expected noise value for a given expression level [14]. See also Figures S2 and S3 and Tables S1 and S2.

(A) Downregulated genes are more variable than upregulated genes. Plots show DM versus expression for up- and downregulated genes (black and purple, respectively) at 6 hr development. Data are shown for different thresholds of fold change (|FC|) in expression level of each gene between 0 and 6 hr, averaged over three replicates. Bin borders are every 500 genes within the entire dataset, starting from a mean of ten counts. Mean and SEM within each bin are shown. Numbers of up- and downregulated genes for each threshold are shown below.

(B) No correlation between RNA stability and gene expression variance. Expression variability in 6-hr cells is plotted against RNA turnover (Pearson r = −0.009). Each dot represents a gene colored by its mean expression level. Degradation units are the ratio of expression before to the expression after 1-hr actinomycin D treatment [7].

versions (Figure 3B, i and v), generated almost exclusively from variance in either size or frequency, did not resemble our data or data from other studies [14, 21].

To what extent can this model framework explain the expression variability of up- and downregulated genes observed in our data? Starting intuitively, transcript levels can be increased by increasing burst size, frequency, or both, so expression increases along the diagonal of a plot of frequency versus size (Figure 3C, i). Increasing burst frequency will reduce the variance in expression (due to time-averaging of noisy events), whereas increasing burst size will increase variance (due to amplification of noisy events). Therefore, the variance in expression is orthogonal to the mean in this parameter space (Figure 3C, ii). It follows that, if expression changes with a constraint on how much frequency or size can vary, this will bias the resulting variance. For example, if genes are regulated predominantly by burst frequency (box in Figure 3C, iii), then this will reduce the variance of the transcript abundance during upregulation.

To test this reasoning, we simulated the changing level of expression of a gene between two developmental time points, by randomly sampling pairs of points from the simulated cloud of genes in Figure 3B (iii). Sampling excluded pairs with less than a 2-fold difference in expression between the points, and it was weighted by the specified change in burst size and frequency. Using these pairs of points provided high and low versions of a simulated gene, giving us the opportunity to look at the overall variance characteristics of genes that have changed their expression level, based on user-defined changes in burst size, frequency, or both.

The simulations revealed differences between regulation dominated by either burst frequency or size. If regulation was equal between size and frequency, the simulation showed no difference in variance between high- and low-expressed genes of a pair (Figure 3D, ii). If regulation was dominated by burst size, the low-expressed partners showed lower variance than the high-expressed partners (Figure 3D, i). In contrast, regulation dominated by burst frequency showed that the low-expressed partners had more variability than the high-expressed partners (Figure 3D, iii).

We can interpret expression changes linking the simulated gene pairs as occurring during developmental time, with the

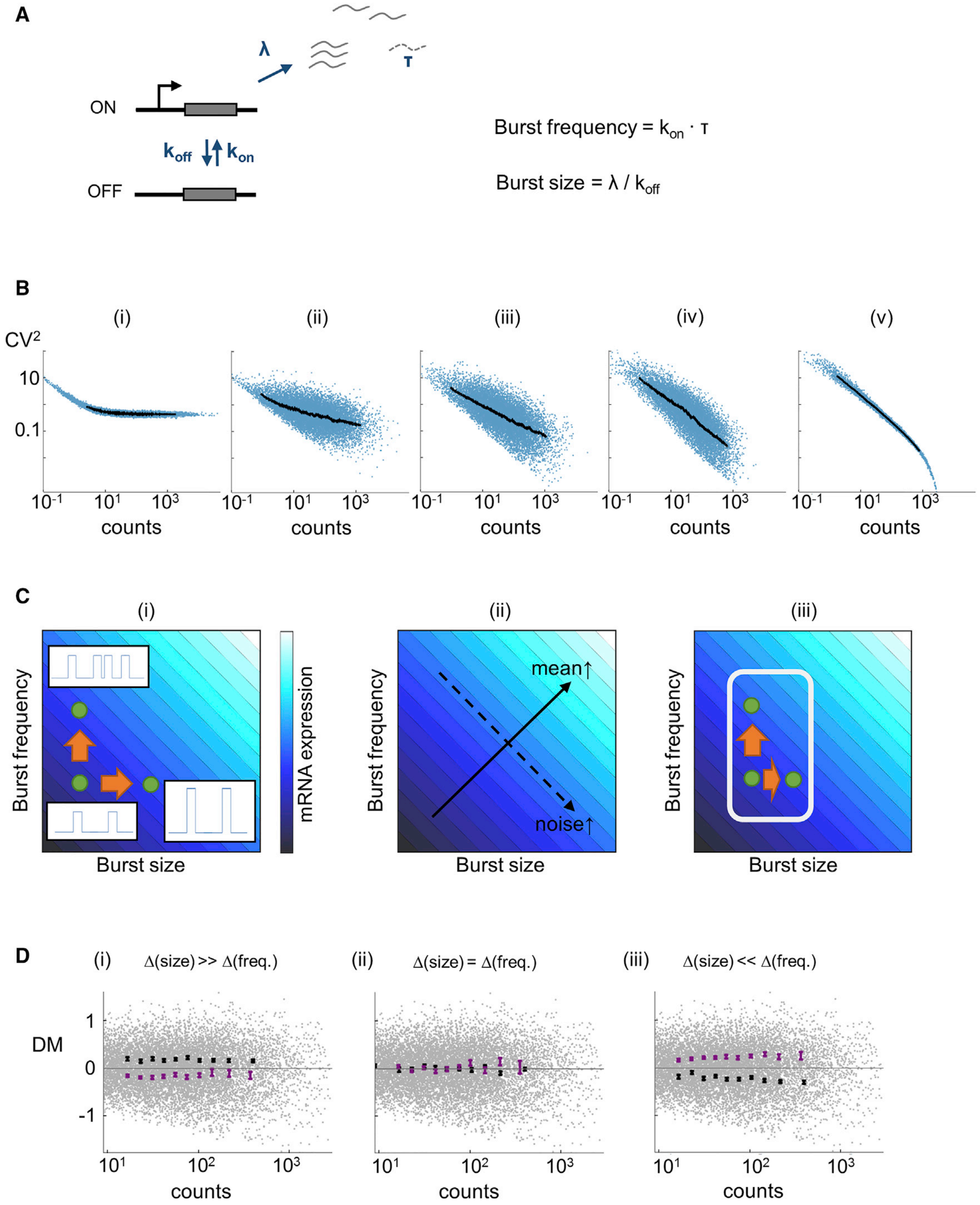

Figure 3. A Simple Model of Transcriptional Dynamics Explains the Global Variance Properties of Up- and Downregulated Genes

(A) Two-state model of transcriptional bursting. The gene toggles between active and inactive states, with rates k_{on} and k_{off}. When active, transcript production occurs at a rate λ with transcript lifetime τ. Transcript burst frequency (the frequency with which the active state occurs) is k_{on}, although in most models k_{on} is scaled by τ. Burst size (the amount of RNA produced per burst) is λ/k_{off}.

(B) Stochastic simulation of transcription based on the model in (A) generates different simulated clouds (i–v) from different pre-set distributions of burst size and frequency (from i, where genes vary predominantly in burst size, to v, where genes vary predominantly in burst frequency, with equal contributions of size and frequency in iii). In (ii)–(iv), where both size and frequency contribute more equally, simulated data more closely resemble the experimental data.

(C) Intuitive explanation of how controlling the burst parameters affects the variance of up- and downregulated genes. (i) Schematic shows mean expression is increased by increasing either burst size or frequency. (ii) Noise increases with burst size and decreases with burst frequency. (iii) Restricting the range of possible sizes and frequencies means the gene can only sample a limited range of values of mean and noise. In the example shown, the gene is mainly regulated via frequency, so an increase in expression favors a decrease in noise.

(legend continued on next page)

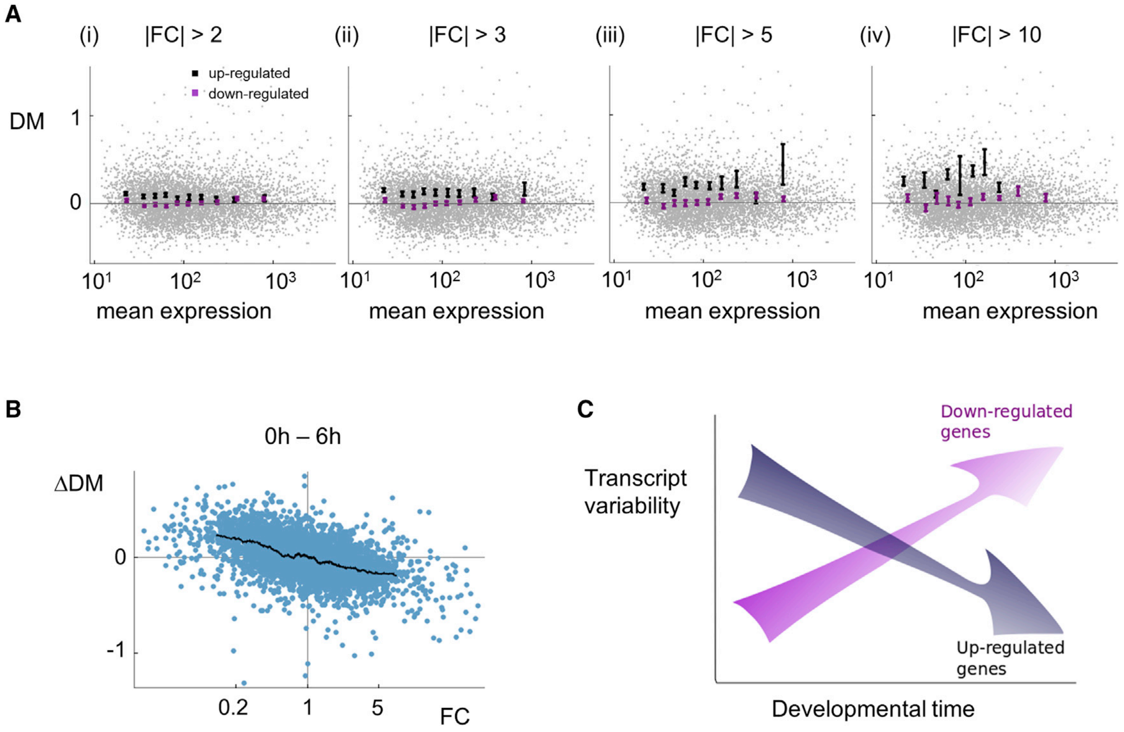

Figure 4. Differentiation-Induced Genes Show Elevated Transcript Variability in Undifferentiated Cells

(A) Plots of variability versus expression level (read counts) for genes that will be up- and downregulated during differentiation (black and purple, respectively) before differentiation onset (0 hr). Bins are defined as in Figure 2. Mean and SEM within bins are shown for different fold-change thresholds.

(B) Negative scaling of change in expression (FC) with the change in transcript variability (ΔDM) during differentiation. Variability falls in upregulated genes and increases in downregulated genes.

(C) Summary. Genes induced during development are initially more variable than genes that will be repressed. Genes that are repressed become more variable than induced genes.

See also Figure S4 and Data S2.

low-expressed partners as the genes that were downregulated and the high-expressed partners as those that were upregulated. If burst size is the predominant source of regulation (Figure 3D, i), we would expect that, in differentiated cells (6 hr), the upregulated genes would be more variable. This was not observed in the experimental data (Figure 2A). If burst frequency is the dominant source of regulation (Figure 3D, iii), we would expect that downregulated genes would be more variable. This scenario matches the experimental data in Figure 2A, implying that the strong contribution of downregulation in generating expression diversity during development can be explained by the regulation of burst frequency.

Imposing down- or upregulation during development onto pairs is arbitrary; pairing only specifies a change in expression governed by a probabilistic set of rules describing the burst parameters. We could equally well interpret the simulated pairs from another point in time, such as the starting point rather than the end point. So we can use the model to predict the *initial* gene expression variance of genes that will be upregulated or downregulated by burst frequency regulation. If we consider the low-expressed partner in Figure 3D (iii) as the gene that will be upregulated by an increase in burst frequency and the high-expressed versions as the genes that will be downregulated by a decrease in burst frequency, we would expect that, in undifferentiated cells (0 hr), the genes that will later be upregulated would initially be more variable. In contrast, genes that will be later downregulated would initially be less variable. Are the experimental data consistent with these predictions?

Analysis of the experimental data indicates that these predictions are valid. Figure 4A shows the plots of transcript level and variance for 0-hr cells, showing genes that will become up- or downregulated during differentiation. The genes that would be upregulated were initially more variable in their expression than those that would be downregulated. The difference is clearer for genes that undergo higher fold changes. For genes changing by 3-fold or more, for the first five bins, the difference was significant at $p < 0.01$ and for the next two bins at $p < 0.05$.

(D) Matching the experimental data in Figure 2A using the two-state model. Lower expressed members of random gene pairs are more variable, if transcriptional output is determined by burst frequency rather than burst size. Shown are the simulations of randomized selections of genes constrained to have >2-fold changes in expression, allowing genes to have more variability in (i) burst size and (iii) frequency; (ii) where frequency and size vary equally. Low-expressed genes from simulated pairs (purple) and their high-expressed partners (black) are shown. Mean and SEM within each bin are shown.

This effect was apparent regardless of the bin size (Figure S4A) and was clear in the unprocessed CV^2 values (Figure S4B). This observation might signify a knee-jerk response, of some undifferentiated cells, to the slightest hint of the differentiation trigger (starvation), and it implies that the system is geared to generate a developmentally advanced sub-population, perhaps with the potential to nucleate subsequent developmental events. In support of this idea, functional enrichment analysis indicates that starvation response genes, in addition to genes from other stress response pathways, were heterogeneously expressed in undifferentiated cells (Data S2). In contrast, the genes that were variably expressed at 6-hr development were strongly enriched for functions in several biosynthetic processes. This may relate to observations that cell fate outcome in *Dictyostelium* can be strongly influenced by the nutritional history of cells [22]. Expression distributions of selected genes following the global variance trends for up- and downregulation are displayed in Figure S4C.

Overall, our data reveal relationships between gene activation and repression and the variability in transcript levels during a developmental transition (Figures 4B and 4C). Transcript levels from genes that will be upregulated are initially more variable than those that will be downregulated. At the end of the developmental transition, transcript levels from genes that were downregulated are more variable than those from genes that were upregulated. The dynamic variability of transcripts can be explained by a simple model, in which genes are regulated by the frequency rather than the magnitude of transcriptional bursts. This view is supported by observations in multiple systems that cell signaling can regulate the frequency rather than the duration of transcriptional responses [18, 23–25]. The model cannot be expected to do justice to the full complexity of transcriptional dynamics within a burst [6], and any effects of cell-cell variation in RNA turnover will also need to be superimposed. However, the analysis represents a good first approximation, which can be compared and adapted to specific molecular effects of activation and repression during cell decision-making.

STAR★METHODS

Detailed methods are provided in the online version of this paper and include the following:

- KEY RESOURCES TABLE
- CONTACT FOR REAGENT AND RESOURCE SHARING
- EXPERIMENTAL MODEL AND SUBJECT DETAILS
- METHOD DETAILS
  - Single-cell RNAseq
  - Stochastic simulation of transcriptional bursting
- QUANTIFICATION AND STATISTICAL ANALYSIS
  - Analysis of read data
  - Bootstrapping
- DATA AND SOFTWARE AVAILABILITY

SUPPLEMENTAL INFORMATION

Supplemental Information includes four figures, two tables, and two datasets and can be found with this article online at http://dx.doi.org/10.1016/j.cub.2017.05.028.

AUTHOR CONTRIBUTIONS

A.M. performed the experiments. V.A., A.M., and J.R.C. analyzed the data. V.A. and A.M.C. performed the modeling. All authors wrote the manuscript.

ACKNOWLEDGMENTS

Single-cell RNA sequencing (RNA-seq) was performed at the Barts and the London Genome Centre. This work was supported by Wellcome Trust Senior Fellowship 202867/Z/16/Z and BBSRC project grant BB/K017004/1 to J.R.C., HFSP long-term fellowship LT000173/2014-L to A.M., and MRC funding (MC_U12266B) to the MRC LMCB University Unit at UCL.

Received: March 22, 2017
Revised: April 28, 2017
Accepted: May 8, 2017
Published: June 8, 2017

REFERENCES

1. Raj, A., and van Oudenaarden, A. (2008). Nature, nurture, or chance: stochastic gene expression and its consequences. Cell *135*, 216–226.
2. Eldar, A., and Elowitz, M.B. (2010). Functional roles for noise in genetic circuits. Nature *467*, 167–173.
3. Rosengarten, R.D., Santhanam, B., Fuller, D., Katoh-Kurasawa, M., Loomis, W.F., Zupan, B., and Shaulsky, G. (2015). Leaps and lulls in the developmental transcriptome of Dictyostelium discoideum. BMC Genomics *16*, 294.
4. Faix, J., Gerisch, G., and Noegel, A.A. (1990). Constitutive overexpression of the contact site A glycoprotein enables growth-phase cells of Dictyostelium discoideum to aggregate. EMBO J. *9*, 2709–2716.
5. Burdine, V., and Clarke, M. (1995). Genetic and physiologic modulation of the prestarvation response in Dictyostelium discoideum. Mol. Biol. Cell *6*, 311–325.
6. Corrigan, A.M., Tunnacliffe, E., Cannon, D., and Chubb, J.R. (2016). A continuum model of transcriptional bursting. eLife *5*, e13051.
7. Muramoto, T., Cannon, D., Gierlinski, M., Corrigan, A., Barton, G.J., and Chubb, J.R. (2012). Live imaging of nascent RNA dynamics reveals distinct types of transcriptional pulse regulation. Proc. Natl. Acad. Sci. USA *109*, 7350–7355.
8. Trapnell, C., Cacchiarelli, D., Grimsby, J., Pokharel, P., Li, S., Morse, M., Lennon, N.J., Livak, K.J., Mikkelsen, T.S., and Rinn, J.L. (2014). The dynamics and regulators of cell fate decisions are revealed by pseudotemporal ordering of single cells. Nat. Biotechnol. *32*, 381–386.
9. Marco, E., Karp, R.L., Guo, G., Robson, P., Hart, A.H., Trippa, L., and Yuan, G.C. (2014). Bifurcation analysis of single-cell gene expression data reveals epigenetic landscape. Proc. Natl. Acad. Sci. USA *111*, E5643–E5650.
10. Setty, M., Tadmor, M.D., Reich-Zeliger, S., Angel, O., Salame, T.M., Kathail, P., Choi, K., Bendall, S., Friedman, N., and Pe'er, D. (2016). Wishbone identifies bifurcating developmental trajectories from single-cell data. Nat. Biotechnol. *34*, 637–645.
11. Richard, A., Boullu, L., Herbach, U., Bonnafoux, A., Morin, V., Vallin, E., Guillemin, A., Papili Gao, N., Gunawan, R., Cosette, J., et al. (2016). Single-Cell-Based Analysis Highlights a Surge in Cell-to-Cell Molecular Variability Preceding Irreversible Commitment in a Differentiation Process. PLoS Biol. *14*, e1002585.
12. Bargaje, R., Trachana, K., Shelton, M.N., McGinnis, C.S., Zhou, J.X., Chadick, C., Cook, S., Cavanaugh, C., Huang, S., and Hood, L. (2017). Cell population structure prior to bifurcation predicts efficiency of directed differentiation in human induced pluripotent cells. Proc. Natl. Acad. Sci. USA *114*, 2271–2276.
13. Petropoulos, S., Edsgärd, D., Reinius, B., Deng, Q., Panula, S.P., Codeluppi, S., Plaza Reyes, A., Linnarsson, S., Sandberg, R., and Lanner, F. (2016). Single-Cell RNA-Seq Reveals Lineage and X

Chromosome Dynamics in Human Preimplantation Embryos. Cell *165*, 1012–1026.
14. Kolodziejczyk, A.A., Kim, J.K., Svensson, V., Marioni, J.C., and Teichmann, S.A. (2015). The technology and biology of single-cell RNA sequencing. Mol. Cell *58*, 610–620.
15. Paulsson, J. (2005). Models of stochastic gene expression. Phys. Life Rev. *2*, 157–175.
16. Kim, J.K., and Marioni, J.C. (2013). Inferring the kinetics of stochastic gene expression from single-cell RNA-sequencing data. Genome Biol. *14*, R7.
17. Raj, A., Peskin, C.S., Tranchina, D., Vargas, D.Y., and Tyagi, S. (2006). Stochastic mRNA synthesis in mammalian cells. PLoS Biol. *4*, e309.
18. Fukaya, T., Lim, B., and Levine, M. (2016). Enhancer Control of Transcriptional Bursting. Cell *166*, 358–368.
19. Stevense, M., Muramoto, T., Müller, I., and Chubb, J.R. (2010). Digital nature of the immediate-early transcriptional response. Development *137*, 579–584.
20. Chubb, J.R., Trcek, T., Shenoy, S.M., and Singer, R.H. (2006). Transcriptional pulsing of a developmental gene. Curr. Biol. *16*, 1018–1025.
21. Goolam, M., Scialdone, A., Graham, S.J., Macaulay, I.C., Jedrusik, A., Hupalowska, A., Voet, T., Marioni, J.C., and Zernicka-Goetz, M. (2016). Heterogeneity in Oct4 and Sox2 Targets Biases Cell Fate in 4-Cell Mouse Embryos. Cell *165*, 61–74.
22. Thompson, C.R., and Kay, R.R. (2000). Cell-fate choice in Dictyostelium: intrinsic biases modulate sensitivity to DIF signaling. Dev. Biol. *227*, 56–64.
23. Cai, L., Dalal, C.K., and Elowitz, M.B. (2008). Frequency-modulated nuclear localization bursts coordinate gene regulation. Nature *455*, 485–490.
24. Cai, H., Katoh-Kurasawa, M., Muramoto, T., Santhanam, B., Long, Y., Li, L., Ueda, M., Iglesias, P.A., Shaulsky, G., and Devreotes, P.N. (2014). Nucleocytoplasmic shuttling of a GATA transcription factor functions as a development timer. Science *343*, 1249531.
25. Corrigan, A.M., and Chubb, J.R. (2014). Regulation of transcriptional bursting by a naturally oscillating signal. Curr. Biol. *24*, 205–211.
26. Mi, H., Huang, X., Muruganujan, A., Tang, H., Mills, C., Kang, D., and Thomas, P.D. (2017). PANTHER version 11: expanded annotation data from Gene Ontology and Reactome pathways, and data analysis tool enhancements. Nucleic Acids Res. *45* (D1), D183–D189.
27. Gillespie, D.T. (1977). Exact stochastic simulation of coupled chemical reactions. J. Phys. Chem. *81*, 2340–2361.
28. Anders, S., and Huber, W. (2010). Differential expression analysis for sequence count data. Genome Biol. *11*, R106.
29. Basu, S., Fey, P., Jimenez-Morales, D., Dodson, R.J., and Chisholm, R.L. (2015). dictyBase 2015: Expanding data and annotations in a new software environment. Genesis *53*, 523–534.
30. Chang, G.S., Noegel, A.A., Mavrich, T.N., Müller, R., Tomsho, L., Ward, E., Felder, M., Jiang, C., Eichinger, L., Glöckner, G., et al. (2012). Unusual combinatorial involvement of poly-A/T tracts in organizing genes and chromatin in Dictyostelium. Genome Res. *22*, 1098–1106.
31. Efron, B. (1994). An Introduction to the Bootstrap (Chapman & Hall).

STAR★METHODS

KEY RESOURCES TABLE

REAGENT or RESOURCE	SOURCE	IDENTIFIER
Critical Commercial Assays		
Cell separation- C1 Integrated Fluidic Circuit chips	Fluidigm	100/5759, 100/5760
cDNA synthesis- Advantage 2 PCR Kit and SMARTer PCR cDNA Synthesis Kit	Clontech	200062
Library preparation- Nextera XT DNA Sample Preparation Kit	Illumina	FC-131-1096
Library preparation- Nextera Index Kit	Illumina	FC-131-1002
Experimental Models: Organisms/Strains		
Dictyostelium AX3 cells with the *rps30* gene engineered to express H2B-Cherry as a nuclear marker	[6, 25]	N/A
Software and Algorithms		
MATLAB R2016a	MathWorks	N/A
Mathematica 10	Wolfram	N/A
R 0.99.486	Open source	N/A
Python 3.5	Open source	N/A
Panther 11	[26]	N/A
Scuba v1.0	[9]	N/A
Wishbone	[10]	N/A
Monocle 1.4.0	[8]	N/A

CONTACT FOR REAGENT AND RESOURCE SHARING

Further information and requests for resources and reagents should be directed to and will be fulfilled by the Lead Contact, Jonathan Chubb (j.chubb@ucl.ac.uk).

EXPERIMENTAL MODEL AND SUBJECT DETAILS

We used *Dictyostelium* AX3 cells (mating type I). The cells had been previously engineered to express a red fluorescent nuclear marker [25]. This marker facilitated validation of single-cell capture for scRNAseq. Cells were cultured in HL5 medium attached to tissue culture dishes [6] as previously described. For development assays, cells were detached from the plastic by pipetting, washed in KK2 phosphate buffer (KPO_4, pH 6.2) and plated on KK2/1.5% agar at a density of 3×10^6 cells per 35mm dish. At the indicated times, cells were detached from the agar by gentle pipetting, and transferred in ice-cold KK2 buffer for cell capture and downstream scRNAseq processing.

METHOD DETAILS

Single-cell RNAseq

For single-cell RNAseq, three replicates of the developmental time series were captured. For each time point, cells were loaded onto Integrated Fluidic Circuit chips (IFC; Fluidigm). We identified capture of multiple cells and empty wells using brightfield illumination, with validation of single-cell capture initially carried out using a genetically encoded red fluorescent nuclear marker. Cell lysis, reverse transcription and cDNA pre-amplification were performed in the C1 Single-Cell Auto Prep IFC using the SMARTer PCR cDNA Synthesis Kit (Clontech) and the Advantage 2 PCR Kit, as specified by the manufacturer (protocol 100-7168 A2). ERCC RNA spike-in control mix (92 transcripts; ThermoFisher) was added to the chambers at a 1:1000 ratio. cDNA was harvested and the libraries were prepared using the Nextera XT DNA Sample Preparation Kit and the Nextera Index Kit (Illumina), according to the manufacturer's recommendations (protocol 100-7168 A2). Libraries from one chip were pooled, and paired-end 75bp (first two replicates) or 25bp (third replicates) sequencing was performed on 4 lanes of an Illumina NextSeq500.

The quality of the reads was assessed using fastqc; a quality control tool for high throughput sequence data (www.bioinformatics.babraham.ac.uk/projects/fastqc/). Paired-end reads were mapped to the *Dictyostelium* genome (version obtained from Gareth Bloomfield, masking the duplication on chromosome 2) using Tophat version 2.0.9 (a spliced read mapper for RNA-seq; built on the mapping program Bowtie). Subsequently, we counted reads for each gene with htseq-count.

Stochastic simulation of transcriptional bursting

The transcription was simulated by a simple two-state model of transcriptional bursting [6], where a gene is either in the OFF state, where no transcription occurs, or in the ON state, where there is a certain probability of polymerase initiation event. The switching between the two states occurs at the rates of k_{on} and k_{off}, with k_{off} being much greater than k_{on} in order for transcription to display a bursting behavior. For these simple purposes, we assume that polymerases are processive, so the rate of RNA production is equal to transcription initiation rate, λ. Once the initiation occurs, the RNA is produced with the offset of dwell time (the time needed for RNA to leave the transcription site - comprised of elongation time and termination time, and set to the physiological value of 120 s [6]). The degradation time, τ, is defined as the lifetime of RNA once it has left the transcription site.

To obtain an amount of RNAs for an individual gene in an individual cell at a certain moment in time, we performed stochastic numerical simulations, in MATLAB, of transcriptional bursting using the Gillespie algorithm [27]. The burst size, or a number of transcripts produced per burst, is defined as λ/k_{off}, and the burst frequency, number of bursts per lifetime of cytoplasmic RNA, is defined as $k_{on} \times \tau$. To mimic the distributions of burst size and burst frequency, τ *and* k_{off} were set to constant values of 1800 *s* and 0.01 s^{-1}, respectively, while λ and k_{on} were sampled from a ($\log_2$)-normal distribution with the mean values of 0.2 s^{-1} and 0.0013 s^{-1}, respectively. Parameters set in this way result in average values of 2.34 bursts per cytoplasmic RNA lifetime and 20 RNAs synthesized during each burst. The RNA lifetime was approximated from RNA decay measurements for around 20 genes, from Northern blot data on actinomycin D treated cells [7]. The value of λ was a medium range estimate of the initiation rate from [6]. The value of k_{off} was approximated from live cell measurements of transcription for a panel of housekeeping and developmental genes, for which the majority had transcription pulses lasting less than 5 min [7]. Our estimate of k_{on} was based upon a compromise between several measurements of transcription pulse interval for different genes [7, 19, 20].

The variance of k_{on} and λ are defined through their levels of contribution to a total variance in the system, as $\sigma^2(\lambda) = c_{size} \times \sigma^2_{tot}$ and $\sigma^2(k_{on}) = c_{freq} \times \sigma^2_{tot}$, with the total variance being $\sigma^2_{tot} = \sigma^2(\lambda) + \sigma^2(k_{on})$. Here, c_{size} and c_{freq} are the user-specified coefficients defining the fraction of the variance contributed by burst size and frequency, respectively. To create different scenarios of how the frequency and size of transcriptional bursts vary across the genome, we set c_{size} and c_{freq} to the following ratios: 1:0, 0.75:0.25, 0.5: 0.5, 0.25:0.75 and 0:1. For each of these scenarios, the simulation generates 200 cells described by 12,000 randomly selected genes. For each gene, we calculated the average number of transcripts, μ, coefficient of variance, CV^2, and the relative noise value, *DM*.

To simulate the process of transcriptional activation or repression, we performed a Gibbs sampling on the aforementioned simulated datasets, in order to retrieve random pairs of genes, which represent one gene either side of the developmental transition. For this purpose, we specified the probability distribution in λ/k_{on} parameter space, reflecting the user-defined probability by which the burst size and the burst frequency can change between genes in a pair. Each gene pair was selected in the following manner:

1. Randomly select the first gene g_{UP}.
2. Repeat the following steps 1000 times:
   a. Before selecting a second gene, reject any that do not fit the two-fold expression change requirement.
   b. Assign the probability to every remaining point based on its distance from the chosen gene g_{UP} in λ/k_{on} parameter space.
   c. Randomly choose one of the points from the previous step, weighted by its probability, as a gene g_{DOWN}.
   d. Find another g_{UP} partner by the same process described in a. to c.
3. Accept the final pair of g_{UP} and g_{DOWN}.

Three different mechanisms of transcriptional regulation were simulated: regulation dominated by changing mostly burst frequency, regulation by changing mostly burst size or regulation by changing both properties equally. In other words, we set the values of $\sigma^2(\lambda)$ and $\sigma^2(k_{on})$ to satisfy one of the following conditions: $\sigma^2(\lambda) << \sigma^2(k_{on})$, $\sigma^2(\lambda) >> \sigma^2(k_{on})$ and $\sigma^2(\lambda) = \sigma^2(k_{on})$. A total of 2000 gene pairs were selected in this manner. Each pair contains genes with mean values at least 2 fold different. In brief, we estimated the total variance (in $\log_2$ space) of the experimental data as 7.3. Generating the simulated gene clouds in Figure 3B divided this variance between $\sigma^2(\lambda)$ *and* $\sigma^2(k_{on})$ in the aforementioned ratios- for example, for the 50:50 split, the SDs for both λ and k_{on} were 1.91, and $1.91^2 = 3.65 = 7.3/2$ (half the total variance). Using the data from the 50:50 simulation, we then set either λ or k_{on} as limiting (SD = 0.3) and the other as non-limiting (SD = 5) for setting the distributions used in the Gibbs pairing.

A key feature of the model is that genes are constrained to lie in a bounded region of burst parameter space, such that there are upper and lower limits to the burst size and frequency. This is biologically reasonable; the transcription machinery must operate within finite physical limits. This feature means that genes of the same mean expression are distributed differently in parameter space (and will have a different variance) depending on whether they are up- or downregulated.

QUANTIFICATION AND STATISTICAL ANALYSIS

Analysis of read data

An average of 3 million reads were generated from each single-cell library. Raw read counts for each gene in each cell at each time point are tabulated in Data S1. We excluded cells with fewer than 500 000 and higher than 7 million reads, to exclude poor or overloaded single-cell libraries. We obtained a total of 433 cells (131 cells at 0h development, 157 cells at 3h and 145 cells at 6h). These cells also satisfied all other cell quality criteria (high number of genes detected in each cell, low percentage of mitochondrial reads and low percentage of low alignment quality reads). Reads from rRNA contaminants were excluded. Read counts of cells within each

replica were normalized using the size factor from the DESeq package [28]. To minimize the impact of technical noise in our analyses, we excluded genes with a mean normalized read count < 10. For comparing the variability of up- and downregulated genes, we considered genes in bins of the same expression range, which further allows us to control for technical noise. As measures of variance we used either squared coefficient of variance $(CV^2 = \sigma^2/\mu^2)$ or DM (distance to median CV^2 value) [14], which accounts for the confounding effects of gene length and mean expression level on the CV^2. Sequences and positional information of upstream intergenic regions were obtained from dictyBase [29]. TATA-containing genes were defined in [30]. RNA degradation rates were obtained from [7]. Data processing was carried out in Mathematica. For pseudotime analyses, Scuba was implemented in MATLAB, Wishbone in Python and Monocle in R. Gene Ontology enrichment analysis was performed with PANTHER Classification System version 11 [26]. GO terms enriched in both heterogeneous and homogeneous gene sets at a specific time point are excluded. Apart from the bootstrapping analysis (below), tests of significance used the non-parametric Mann-Whitney test, in Mathematica. Statistical details are provided in the relevant figure legends and manuscript text.

Bootstrapping

To estimate the variabilities of the calculated mean DM values of up- and downregulated genes in each bin, we performed bootstrap sampling as described [31] in Mathematica. For each bin we repeated the following procedure 10 000 times:

1. Separately resample, with replacement, upregulated and downregulated genes.
2. In each resampled set, normalize each observation's value (DM_x) to the same mean:

$$\widetilde{DM}_{x \in up} = DM_x - \overline{DM}_{up} + \overline{DM}_{up \cup down}$$

or

$$\widetilde{DM}_{x \in down} = DM_x - \overline{DM}_{down} + \overline{DM}_{up \cup down},$$

depending on whether the observation belongs to the up- or downregulated resampled set. In this way, we transform each sample to have a mean equal to the overall mean of the population and so generate the H_0 hypothesis.

3. Perform the Student's t test between the distributions of $\widetilde{DM}_{up}$ and $\widetilde{DM}_{down}$ to get a t-value, t_i.

After collecting the n = 10 000 t_i values, we calculate the probability of *DM* values of up- and downregulated genes to be significantly different by chance as:

$$p(H_0) = \frac{1 + \sum_{i=1}^{n} (t_i > t)}{1 + n},$$

with t being the original sample t-value.

DATA AND SOFTWARE AVAILABILITY

Raw read count data from the single-cell RNAseq is provided as Data S1. The MATLAB code used for the simulations is available at http://www.ucl.ac.uk/lmcb/sites/default/files/Simulation_2017.zip.

Current Biology, Volume *27*

Supplemental Information

Generation of Single-Cell Transcript Variability by Repression

Vlatka Antolović, Agnes Miermont, Adam M. Corrigan, and Jonathan R. Chubb

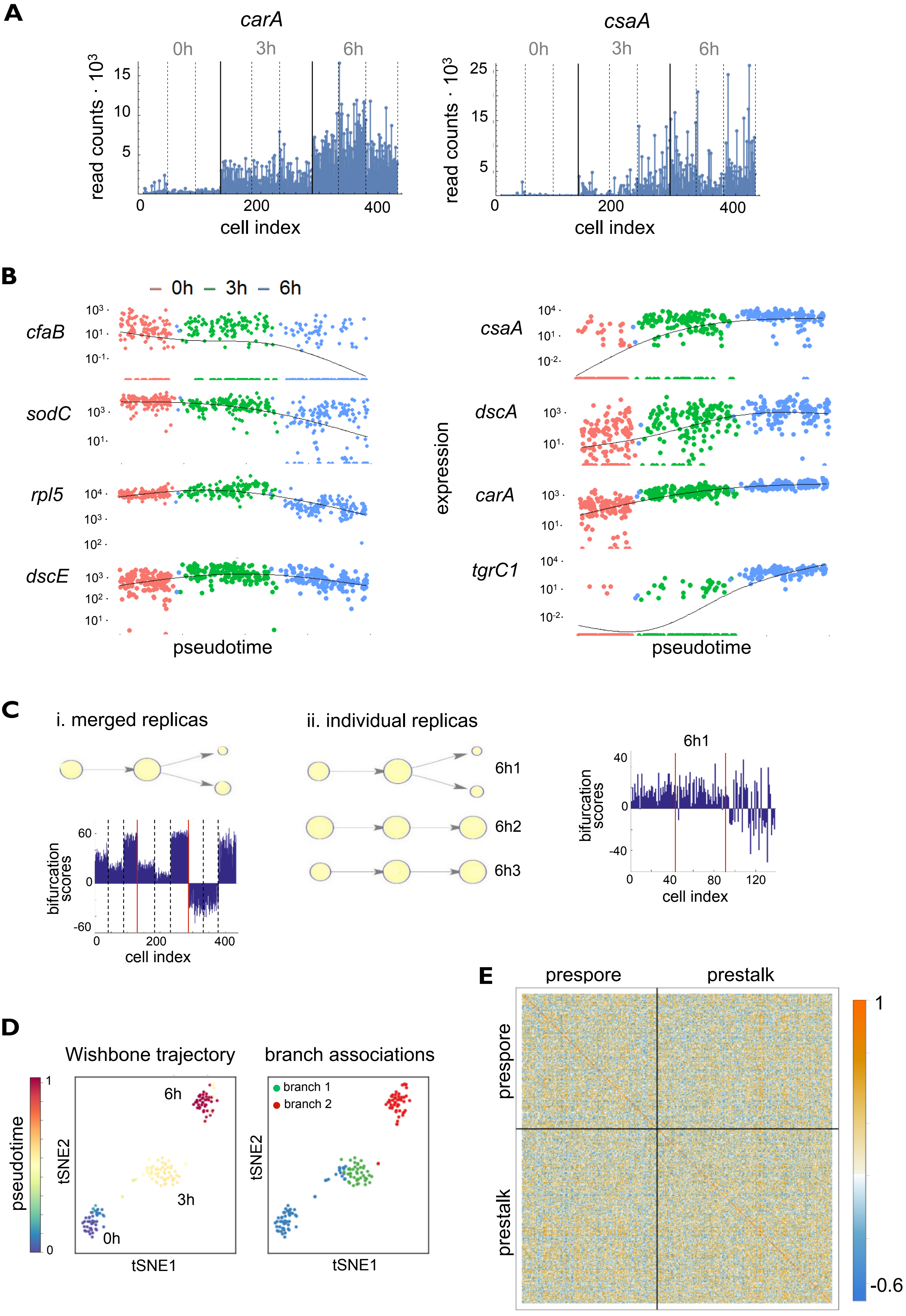
A
carA
csaA
0h
3h
6h
read counts · 10³
cell index
B
0h 3h 6h
cfaB
sodC
rpl5
dscE
csaA
dscA
carA
tgrC1
expression
pseudotime
C
i. merged replicas
ii. individual replicas
6h1
6h2
6h3
bifurcation scores
cell index
D
Wishbone trajectory
branch associations
pseudotime
branch 1
branch 2
tSNE1
tSNE2
E
prespore
prestalk
1
-0.6

Figure S1 . Testing single cell expression analyses and cell trajectories. Related to Figure 1.

A) Expression profiles of known developmental marker genes, *carA* and *csaA* throughout development. Plots show cell read counts from each developmental time point, with the three developmental time points separated by solid vertical gridlines and each replica separated by dotted vertical gridlines. **B)** To test the validity of Monocle, we extracted the changing expression levels of well-studied developmental marker genes in pseudotime. Each dot represents a cell coloured by its developmental time point (red (0h), green (3h) and blue (6h)) and ordered along the x-axis by its assigned pseudotime, and y-axis by the expression level of the indicated gene. The black line shows a smoothed running median of gene expression levels. There is good agreement between these pseudotime-series' and published data. **C)** Bifurcation trees generated by SCUBA using either cells from all 3 replicates (i), or cells from separated replicates (ii). Bottom panel for (i) shows bar plots of the scores for each cell on the bifurcation axis (above the axis is one branch, below the axis is another). The three developmental time points are separated by vertical red lines, and replicates by vertical dotted lines. The bifurcation in i) detects the differences between replicas, not cells within a replica. Only 1/3 replicates shows a bifurcation at 6h. The bifurcation plot for (ii) is only for the replicate (6h1) showing a bifurcation. **D)** Wishbone trajectory and branch association are visualized on tSNE maps. The first two diffusion components are used, based on their highest contribution to overall population variance. When branching is imposed, the 3h and 6h time points are identified as two different developmental cell fates. The use of some higher order diffusion components can result in branching at 6h, although this result is inconsistent between replicas and may be driven mostly by the variability in the developmental progress of the early aggregative cells. **E)** No clear patterns of correlation within lineage specific genes in single cells. Correlation heatmap for the 6h timepoint (replicates pooled) split into prespore and prestalk genes. We selected cell-type specific genes from the intersection between the data sets of [S1] and DictyExpress [S2] with $|\log_2(FC)| > 1$, $-\log_{10}(FDR) > 1$ and a maximum expression level > 100.

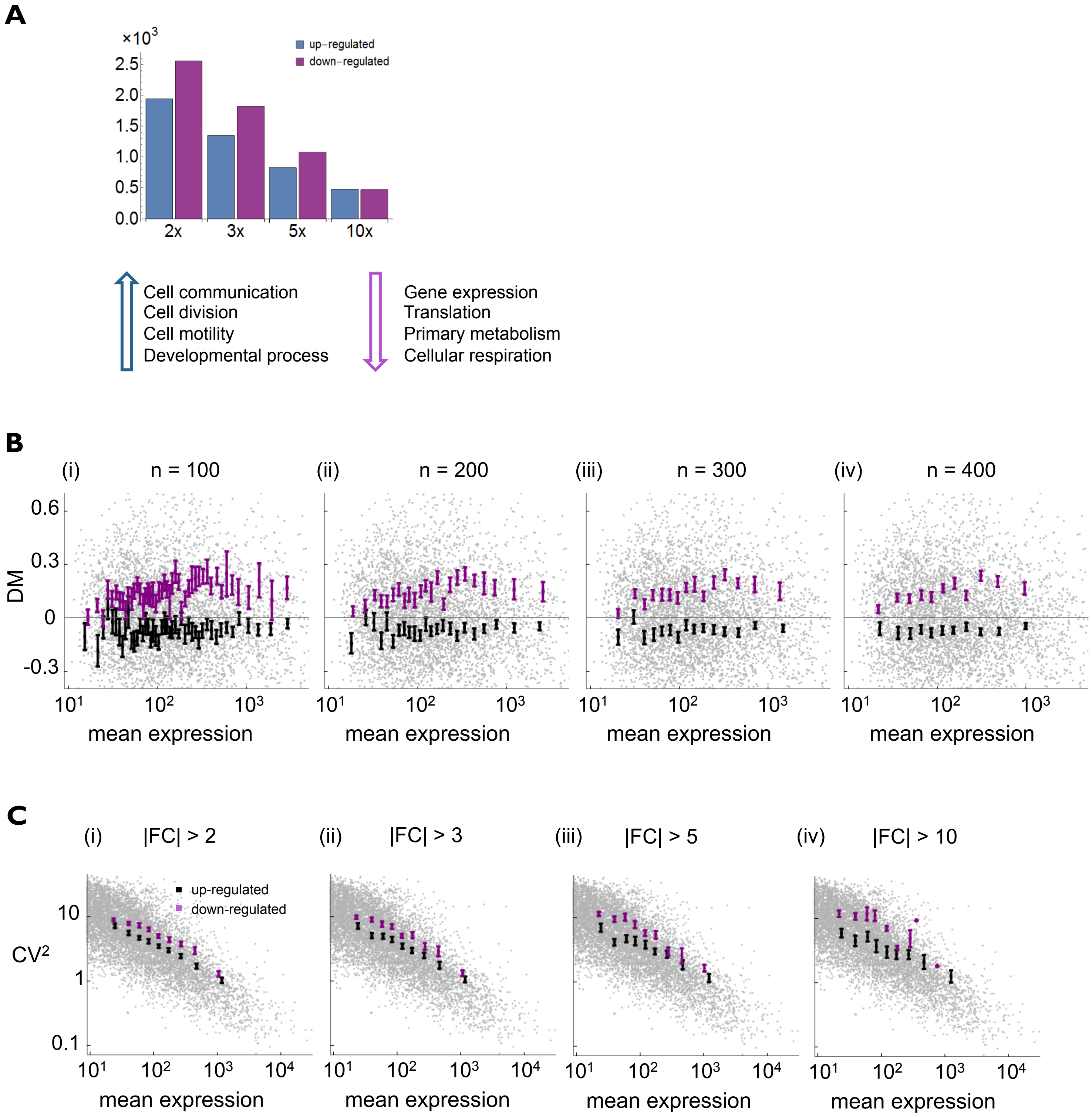
A
up−regulated
down−regulated
Cell communication
Cell division
Cell motility
Developmental process
Gene expression
Translation
Primary metabolism
Cellular respiration
B
(i) n = 100
(ii) n = 200
(iii) n = 300
(iv) n = 400
DM
mean expression
C
(i) |FC| > 2
(ii) |FC| > 3
(iii) |FC| > 5
(iv) |FC| > 10
CV2
mean expression

Figure S2. Effects of gene down-regulation on transcript variability. Related to Figure 2.

A) More genes are repressed than activated as cells differentiate. Around 25 % more genes are down-regulated than up-regulated as cells progress from the undifferentiated state (0h) to the onset of multicellularity (6h). The numbers of up- and down-regulated genes (blue and purple, respectively) are shown for the indicated different thresholds of fold change. Data are averaged from 3 experimental replicates. **B)** Testing the independence of chosen bin size on the observation that down-regulated genes in aggregative cells (6h) are more variable than up-regulated genes. Plots of DM (variability) versus expression level (read counts) for up- and down-regulated genes (black and purple, respectively) for the threshold of fold change $|FC| > 2$. Data are shown for the bin borders defined as every 100 (i), 200 (ii), 300 (iii) or 400 (iv) genes from the whole data set, starting from mean expression value of 10. Genes that were up- and down-regulated between 0h (undifferentiated cells) and 6h development (aggregating cells) are identified, and their mean DM values and standard errors are shown within each bin at the 6h timepoint. **C)** Down-regulated genes show more transcript variability than up-regulated genes. These data are the same as in Figure 2A, using CV^2 values, rather than DM, which has been corrected for transcript size. For $|FC| > 2$, the first and last bins were significant at $p<0.05$. All other bins were significant at $p<0.001$.

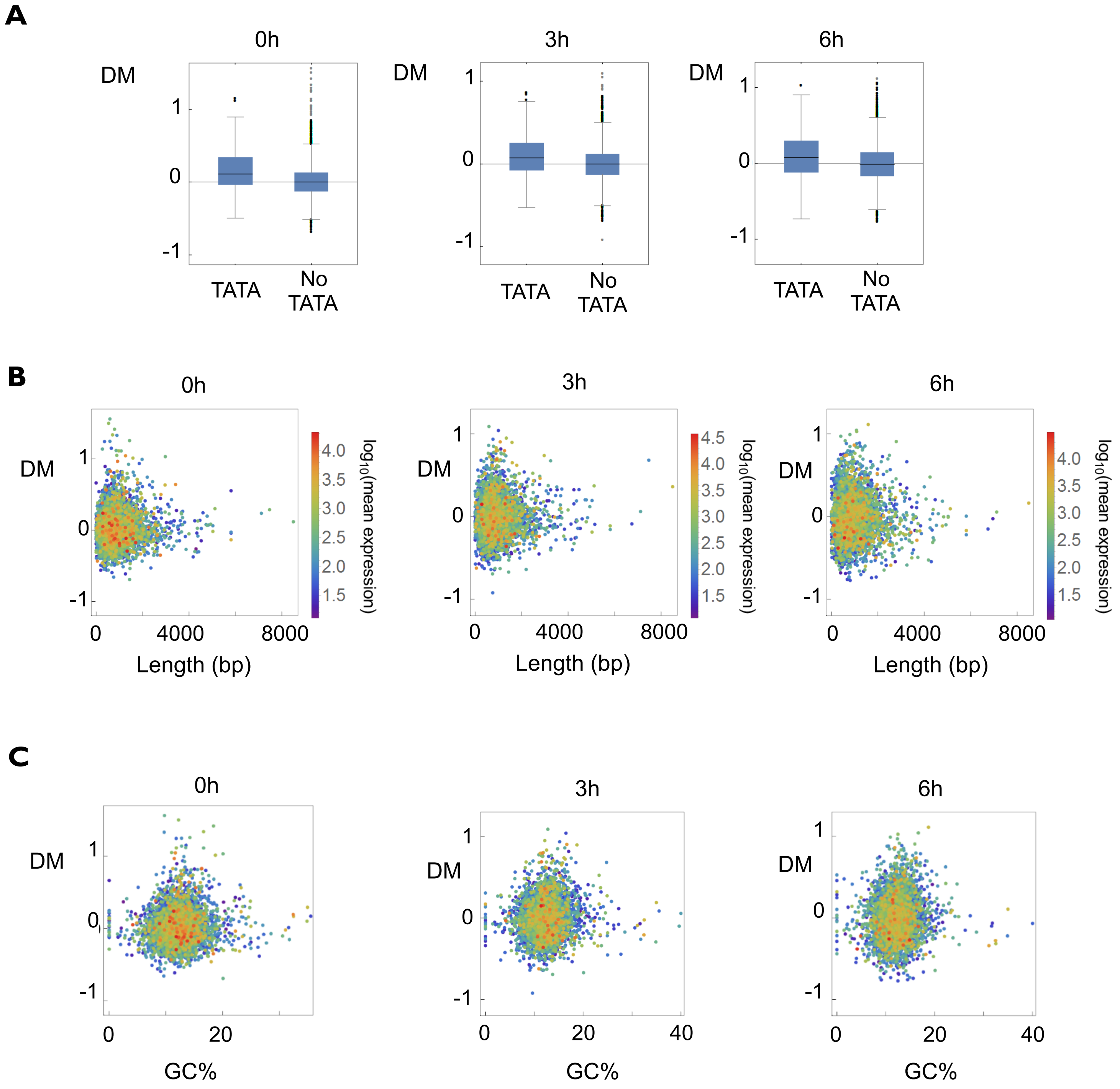

A
0h
3h
6h
DM
1
0
-1
TATA
No TATA
B
0h
3h
6h
DM
1
0
-1
0
4000
8000
Length (bp)
log10(mean expression)
4.5
4.0
3.5
3.0
2.5
2.0
1.5
C
0h
3h
6h
DM
1
0
-1
0
20
40
GC%

Figure S3. Promoter features and transcript diversity. Related to Figure 2.

A) Genes with an identified TATA box in the promoter region show higher expression variability. However, this effect is dependent upon correcting DM values for transcript size. TATA genes tend to be shorter than non-TATA genes in *Dictyostelium*, and in uncorrected CV^2 data, no difference is seen. Distributions of expression variability in the population of 0h cells (0h, Mann-Whitney p= 3.9×10^{-11}), 3h differentiated cells (Mann-Whitney p= 9.1×10^{-6}) and the aggregative cells (6h; p= 3.4×10^{-6}) for genes with and without TATA boxes are shown. **B)** No correlation between promoter length and gene expression variability. DM plotted against length of upstream intergenic region for all three time points. Pearson r values are: 0.06 (0h), 0.03 (3h) and -0.02 (6h). Each dot represents a gene coloured by its mean expression level. **C)** No correlation between promoter GC content and gene expression variability at any time point during early development. Pearson r values: 0.08 (0h), 0.07 (3h) and 0.07 (6h). Each dot represents a gene coloured by its mean expression level.

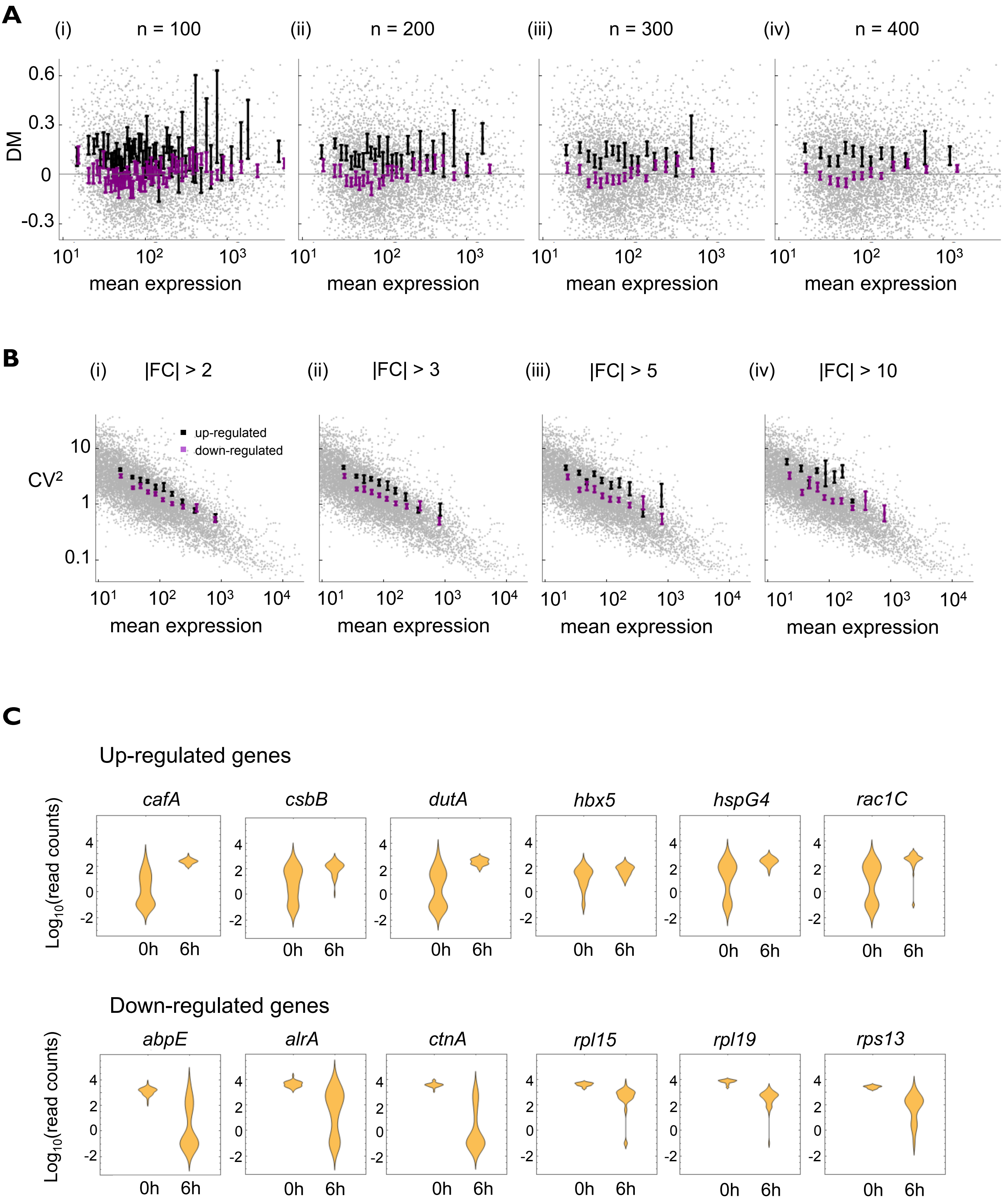

A
(i) n = 100
(ii) n = 200
(iii) n = 300
(iv) n = 400
DM
0.6
0.3
0
-0.3
10^1
10^2
10^3
mean expression
B
(i) |FC| > 2
(ii) |FC| > 3
(iii) |FC| > 5
(iv) |FC| > 10
up-regulated
down-regulated
CV^2
10
1
0.1
10^4
C
Up-regulated genes
cafA
csbB
dutA
hbx5
hspG4
rac1C
Log10(read counts)
4
2
0
-2
0h
6h
Down-regulated genes
abpE
alrA
ctnA
rpl15
rpl19
rps13

Figure S4. Robustness of transcript variability measurements. Related to Figure 4.

A) Testing the independence of chosen bin size on the observation that genes in undifferentiated cells (0h) that will be down-regulated are less variable than genes that will be up-regulated. Plots of DM (variability) versus expression level (read counts) for genes that will be up- and down-regulated during differentiation (black and purple, respectively) before differentiation onset (0h). Data are shown for the bin borders defined as every 100 (i), 200 (ii), 300 (iii) or 400 (iv) genes from the whole data set, starting from mean expression value of 10. **B)** Transcripts that will be up-regulated in development are initially more variable. These data are the same as in Figure 4A, using normalised CV^2 values, rather than DM, which has been corrected for transcript size. For $|FC| > 2$, the first 8 bins showed a significant difference at $p<0.001$. **C)** Transcript count distributions of selected genes representing the global transcriptome trends. Upper row- genes up-regulated during differentiation showing strongly reduced variability. Genes are *cafA* (calcium binding), *csbB* (cell adhesion), *dutA* (ncRNA), *hbx5* (homeobox transcription factor), *hspG4* (heat shock protein), *rac1C* (small GTPase). Bottom- genes down-regulated during differentiation with increased variability. Genes are *abpE* (actin binding protein), *alrA* (aldehyde reductase), *ctnA* (quorum sensing), *rpl15, rpl19 and rps13* (all ribosomal proteins).

GENE LENGTH								
Fold change threshold	All genes		Up-regulated			Down-regulated		
	**Median [IQR] (bp)**	**n**	**Median [IQR] (bp)**	**n**	**p-value**	**Median [IQR] (bp)**	**n**	**p-value**
**2**	1482 [878, 2446]	11244	1861 [1143, 3031]	1889	9.4×10^{-38}	1411 [891, 2191]	2509	9.1×10^{-3}
**3**			1839 [1106, 3012]	1315	2.6×10^{-23}	1356 [864, 2130]	1788	2.3×10^{-5}
**5**			1719 [993, 2813]	798	2.6×10^{-7}	1344 [849, 2064]	1060	3.6×10^{-5}
**10**			1689 [930, 2761]	460	2.1×10^{-3}	1365 [892, 2076]	464	1.9×10^{-2}
INTERGENIC DISTANCE								
Fold change threshold	All genes		Up-regulated			Down-regulated		
	**Median [IQR] (bp)**	**n**	**Median [IQR] (bp)**	**n**	**p-value**	**Median [IQR] (bp)**	**n**	**p-value**
**2**	678 [413, 1058]	11244	853 [517, 1325]	1889	1.7×10^{-34}	618 [387, 944]	2509	2.2×10^{-9}
**3**			871 [523, 1379]	1315	2.9×10^{-29}	620 [390, 945]	1788	7.5×10^{-7}
**5**			891 [559, 1382]	798	5.7×10^{-25}	623 [395, 981]	1060	2.0×10^{-3}
**10**			921 [585, 1423]	460	1.9×10^{-19}	640 [390, 978]	464	3.4×10^{-2}

Table S1. Comparing expression behaviour with gene length and intergenic distance. Related to Figure 2.

Distributions of gene length and intergenic distance (acquired from dictyBase) in the sets of genes detected as up- and down-regulated at different fold change thresholds are compared with the distribution of gene length and intergenic distance throughout the *Dictyostelium* genome. Mann-Whitney test *p*-values are shown. Interquartile ranges (IQR) shown in brackets.

Fold change threshold	All genes		Up-regulated			Down-regulated		
	**Median [IQR]**	**n**	**Median [IQR]**	**n**	**p-value**	**Median [IQR]**	**n**	**p-value**
**2**	1.29 [0.64, 1.96]	13210	1.48 [0.98, 2.19]	1916	1.8×10^{-28}	1.42 [1.00, 1.94]	2496	4.4×10^{-21}
**3**			1.44 [0.90, 2.20]	1335	7.5×10^{-17}	1.42 [0.99, 1.96]	1771	8.9×10^{-17}
**5**			1.35 [0.82, 2.11]	816	1.8×10^{-5}	1.43 [1.01, 2.01]	1041	3.3×10^{-14}
**10**			1.30 [0.70, 2.01]	471	0.13	1.53 [1.04, 2.12]	450	7.6×10^{-11}

Table S2. Comparing expression behaviour with RNA turnover. Related to Figure 2.

Distributions of RNA turnover for the sets of genes detected as up- and down-regulated at different fold change thresholds are compared with the distribution of RNA turnover throughout the *Dictyostelium* genome. Mann-Whitney test *p*-values are shown. Interquartile ranges shown in brackets. RNA turnover (degradation) expressed as ratio of expression before and after 1h treatment with actinomycin D.

Supplemental References

S1. Parikh, A., Miranda, E.R., Katoh-Kurasawa, M., Fuller, D., Rot, G., Zagar, L., Curk, T., Sucgang, R., Chen, R., Zupan, B., et al. (2010). Conserved developmental transcriptomes in evolutionarily divergent species. Genome biology *11*, R35.

S2. Rot, G., Parikh, A., Curk, T., Kuspa, A., Shaulsky, G., and Zupan, B. (2009). dictyExpress: a Dictyostelium discoideum gene expression database with an explorative data analysis web-based interface. BMC bioinformatics *10*, 265.
